# Impact of Gene Repression on Biofilm Formation of *Vibrio cholerae*

**DOI:** 10.3389/fmicb.2022.912297

**Published:** 2022-06-02

**Authors:** Joao P. Pombo, Stephan P. Ebenberger, Anna M. Müller, Heimo Wolinski, Stefan Schild

**Affiliations:** ^1^Institute of Molecular Biosciences, University of Graz, Graz, Austria; ^2^Field of Excellence Biohealth – University of Graz, Graz, Austria; ^3^BioTechMed Graz, Graz, Austria

**Keywords:** *Vibrio cholerae*, biofilm, repressed genes, reporter system, regulation, recombination based, resolvase

## Abstract

*Vibrio cholerae*, the etiological agent of cholera, is a facultative intestinal pathogen which can also survive in aquatic ecosystems in the form of biofilms, surface-associated microbial aggregates embedded in an extracellular matrix, which protects them from predators and hostile environmental factors. Biofilm-derived bacteria and biofilm aggregates are considered a likely source for cholera infections, underscoring the importance of *V. cholerae* biofilm research not just to better understand bacterial ecology, but also cholera pathogenesis in the human host. While several studies focused on factors induced during biofilm formation, genes repressed during this persistence stage have been fairly neglected. In order to complement these previous studies, we used a single cell-based transcriptional reporter system named TetR-controlled recombination-based in-biofilm expression technology (TRIBET) and identified 192 genes to be specifically repressed by *V. cholerae* during biofilm formation. Predicted functions of in-biofilm repressed (ibr) genes range from metabolism, regulation, surface association, transmembrane transport as well as motility and chemotaxis. Constitutive (over)-expression of these genes affected static and dynamic biofilm formation of *V. cholerae* at different stages. Notably, timed expression of one candidate in mature biofilms induced their rapid dispersal. Thus, genes repressed during biofilm formation are not only dispensable for this persistence stage, but their presence can interfere with ordered biofilm development. This work thus contributes new insights into gene silencing during biofilm formation of *V. cholerae*.

## Introduction

*Vibrio cholerae* is a facultative bacterial pathogen and the causative agent of cholera, an acute life-threatening diarrheal disease which affects approximately 2.9 million people per year and causes an estimated 95,000 deaths every year in 69 countries where the disease is endemic (Ali et al., 2015). Virulent *V. cholerae* enters the body when ingested together with contaminated food or water and then proceeds to colonize the small bowel and induce illness upon conditional expression of many well-known virulence factors, such as the cholera toxin and the toxin co-regulated pilus (TCP). Treatment of piped water is effective in preventing the risk of the water-borne disease. However, in low-income countries where cholera is endemic, potable water is sometimes not available, hygiene is poor and treatments or medical care are often insufficient (Harris et al., 2012).

In-between epidemic outbreaks, *V. cholerae* survives in coastal and estuarine aquatic environments, where it mostly exists in the form of sessile microbial communities embedded in an exopolymeric matrix, called biofilms (Schulze et al., 2021). Bacterial biofilms are formed through attachment to biotic or abiotic surfaces and protect enclosed bacteria from microbial predators, bacteriophages, toxic chemicals, and desiccation, while also serving as platforms for accumulation of nutrients and horizontal gene transfer (Flemming et al., 2016). Throughout its life cycle, *V. cholerae* shuttles between aquatic ecosystems and the human host environment, and thus needs to integrate an array of external signals in order to adapt accordingly. Biofilms seem to have a crucial role in this transition, as cells in a biofilm display markedly higher *in vivo* fitness and are more primed for intestinal colonization when compared to their planktonic counterparts (Tamayo et al., 2010; Gallego-Hernandez et al., 2020). Moreover, ingestion of floating biofilm aggregates seems to be a high risk factor for cholera, as simply removing particles larger than 20 μm from contaminated waters prior to use reduced the incidence of cholera by almost 50% (Colwell et al., 2003), underlining the importance of biofilms not only to better understand the ecology of *V. cholerae* and how it persists and behaves during inter-epidemic periods, but also to gain knowledge on the mechanisms of infection and disease, for improved clinical management.

Over the past 30 years, knowledge about the regulatory mechanisms, biological functions, and biochemical composition of *V. cholerae* biofilms substantially increased. Biofilm formation can be generally divided into three stages: attachment, maturation, and dispersal. In *V. cholerae*, the attachment phase initiates upon surface contact. This is facilitated by flagellum-driven rotating motility across the surface, which maximizes contact between the mannose-sensitive hemagglutinin (MSHA) type IV pilus and the surface in order for the cells to find optimal adhesion spots and irreversibly attach themselves (Utada et al., 2014). In addition, the structural matrix protein Bap1 seems to be also relevant for initial attachment (Berk et al., 2012), as well as the c-di-GMP-regulated adhesins FhrA and CraA (Kitts et al., 2019). Furthermore, the type IV pilus TCP, which is necessary for adherence to the small bowel epithelium, has been implicated to facilitate attachment to chitinous substrates found in the aquatic ecosystem (Reguera and Kolter, 2005).

Upon attachment, *V. cholerae* proliferates in a sessile state while simultaneously secreting the components of the extracellular matrix. This matrix is mainly composed of the *Vibrio* polysaccharide (VPS), the structural proteins RbmA, RbmC, and Bap1, and extracellular DNA (Yildiz and Schoolnik, 1999; Fong and Yildiz, 2007; Absalon et al., 2011; Seper et al., 2011). VPS is the most prevalent component of the extracellular biofilm matrix, accounting for over 50% of its total mass (Yildiz et al., 2014). Expression of most biofilm-relevant gene clusters, such as *vps* gene clusters, is negatively regulated by quorum sensing (Hammer and Bassler, 2003) and induced by high levels of the second messenger bis-(3′-5′)-cyclic dimeric guanosine monophosphate (c-di-GMP; Lim et al., 2006; Krasteva Petya et al., 2010; Hsieh et al., 2018).

When biofilms mature, cells disperse from the upper layers and return to the planktonic state to colonize other environments. This process is less well understood, but recent efforts have revealed factors implicated in biofilm dispersal in *V. cholerae*, namely c-di-GMP signaling-related proteins, matrix digestion enzymes, flagellar motility modulators, two-component regulators, and extracellular nucleases (Seper et al., 2011; Bridges et al., 2020).

In summary, many molecular factors are known today whose expression and activity are necessary for proper biofilm development. Most of the reports analyzing expression profiles in *V. cholerae* biofilms relied on transcriptome analyses. However, microarray or RNAseq studies have to deal with two main limitations: (i) the consequences of averaging heterogeneity in the bacterial population and (ii) the restriction to a snapshot analysis (Beloin and Ghigo, 2005; An and Parsek, 2007; Stewart and Franklin, 2008). Any unique patterns of gene expression in specific regions in a biofilm or at specific time-points during biofilm formation might be lost. Most likely cells in the biofilm are in different metabolic states, and the average RNA levels per cell can vary drastically. Transcriptional profiling on RNA levels might be skewed toward the fast-growing population with higher RNA levels. Not surprisingly, three independent transcriptome studies on genes expressed in *Escherichia coli* K-12 biofilms share only two genes in common (Schembri et al., 2003; Beloin et al., 2004; Ren et al., 2005). Thus, alternative technologies, which can overcome at least some of these limitations, are required to unravel new insights in biofilm physiology.

Single-cell based reporter gene systems are useful strategies to explore gene expression in heterogeneous environments like biofilms but have been under-used (Beloin and Ghigo, 2005; An and Parsek, 2007; Stewart and Franklin, 2008). Recombination-based systems are capable of detecting gene deregulation in sub-populations according to their spatial and temporal expression and could therefore reveal novel insights into the biofilm physiology (Angelichio et al., 1999, 2004; Schild et al., 2007; Seper et al., 2014; Cakar et al., 2018).

In addition, most studies performing transcriptome analyses have mainly focused on static biofilms of *V. cholerae*, demonstrating a strict dependence of biofilm formation on the *vps* and quorum sensing genes, which are essential for exopolysaccharide formation and cell–cell signaling, respectively. Static biofilm models are generally performed in enclosed systems, such as test tubes or microtiter plates allowing high-throughput analyses, but result in accumulation of metabolic end products and provide limited supply of nutrients over time. In contrast, dynamic biofilm models using flow cell systems allow a continuous nutrient supply over time (Sternberg and Tolker-Nielsen, 2006), which might reflect a more natural situation, since in the aquatic environment *V. cholerae* is also exposed to flows and currents. Dynamically grown biofilms of O1 El Tor were also reported to be VPS- and quorum sensing-independent (Müller et al., 2007). Intrigued by this observation, a recombination-based screen to identify genes induced during biofilm formation identified several candidates to be exclusively induced in biofilm conditions, and respective mutants exhibited altered phenotypes only in one condition (Seper et al., 2014). This highlights a discrepancy between static and dynamic biofilm formation.

In this work, we have adapted a TetR-controlled resolvase-based single-cell reporter system, previously used to identify *in vivo* repressed genes of *V. cholerae* (Cakar et al., 2018), to now be capable to screen in-biofilm repressed (ibr) genes. So far, little attention has been given to transcriptional silencing during biofilm formation. Such in-biofilm repressed genes might encode factors, which are simply not required during the sessile persistence state. In contrast, their expression could also interfere in biofilm formation and repression is mandatory to allow regular biofilm development. The screen presented herein identified 192 unique in-biofilm repressed genes. Of these, nine were comprehensively analyzed for their impact on biofilm formation and biofilm-associated phenotypes. In general, the vast majority of these nine ibr genes markedly affected static biofilm formation upon constitutive (over)-expression, while most deletion mutants showed no or only minor changes. Further analyses revealed that constitutive (over)-expression of ibr genes can impact biofilm development at different stages. This study therefore highlights the importance of gene repression of *V. cholerae* during biofilm formation.

## Materials and Methods

### Bacterial Strains and Growth Conditions

Bacterial strains and plasmids used in this study are listed in Table 1; oligonucleotides are listed in Table 2. *Vibrio cholerae* AC53 was used as the wild type (WT) strain and is a spontaneous streptomycin-resistant (Sm^R^) derivative of the O1 El Tor Ogawa clinical isolate E7946 (Miller et al., 1989). All *V. cholerae* mutant strains used in this study are derivatives of AC53. *Escherichia coli* strains DH5αλ*pir* and SM10*λpir* were used for genetic manipulations. Unless stated otherwise, strains were cultivated in lysogeny broth (LB) or on LB agar plates with aeration at 37°C. If required, antibiotics and other supplements were used in the following final concentrations: streptomycin (Sm), 100 μg/ml; ampicillin (Ap), 100 μg/ml, or 50 μg/ml in combination with other antibiotics, chloramphenicol (Cm), 2 μg/ml; kanamycin (Km), 50 μg/ml; arabinose (Ara), 0.2% (w/v); glucose (Glc), 0.2% (w/v; for overnight cultures), or 0.02% (w/v; for biofilm dispersal assays); sucrose (Suc), 10% (w/v).

**Table 1:** Bacterial strains and plasmids used in this study.

Strain or plasmid	Genotype/resistance/description	References
*Escherichia coli*		
DH5αλ*pir*	F^−^Φ80*ΔlacZ*Δ*M15*Δ(*argF lac*)*U169 deoR recA1 endA1 hsdR17* (r_K_^−^m_K_^+^) *supE44 thi-1 gyrA69 relA1*, *λpir*R6K	Platt et al., 2000
SM10λ*pir*	thi thr leu tonA lacY supE recA::RPA-2-Te::Mu λpirR6K, Km^R^	Simon et al., 1983
*V. cholerae*		
WT	AC53, wild type *V. cholerae* strain serogroup: O1; biotype: El Tor; serotype: Ogawa; spontaneous Sm^r^ mutant of E7946; clinical isolate from Bahrain 1978; *hapR*^+^, Sm^R^; used for previous immunization studies (Schild et al., 2008, 2009; Bishop et al., 2010, 2012; Leitner et al., 2013)	Miller et al., 1989
*irgA*::*tpc*	Insertion of *tetR-phoA-cat* downstream of *irgA* in WT, Sm^R^, Cm^R^	Cakar et al., 2018
ΔVC0178	Deletion of VC0178 in WT, Sm^R^	This study
ΔVC0512	Deletion of VC0512 in WT, Sm^R^	This study
ΔVC0845	Deletion of VC0845 in WT, Sm^R^	This study
ΔVC0998	Deletion of VC0998 in WT, Sm^R^	This study
ΔVC1289	Deletion of VC1289 in WT, Sm^R^	This study
ΔVCA0281	Deletion of VCA0281 in WT, Sm^R^	This study
ΔVCA0658	Deletion of VCA0658 in WT, Sm^R^	This study
ΔVCA0773	Deletion of VCA0773 in WT, Sm^R^	This study
ΔVCA0988	Deletion of VC0988 in WT, Sm^R^	This study
*vpsA*::*phoA*	Insertion of *phoA* directly downstream of the *vpsA* locus in WT, Sm^R^	This study
*Plasmids*		
pBK	pBAD18-Kan, arabinose-inducible, Km^R^	Guzman et al., 1995
pCVD442	ori6K, mobRP4, *sacB*, Ap^R^	Donnenberg and Kaper, 1991
pUC19	pUC6 backbone with M13mp19-derived multiple cloning site in reverse orientation, high copy number, Ap^R^	Norrander et al., 1983
pBK-VC0178	VC0178 of WT in pBAD18-Kan, Km^R^	This study
pBK-VC0512	VC0512 of WT in pBAD18-Kan, Km^R^	This study
pBK-VC0845	VC0845 of WT in pBAD18-Kan, Km^R^	This study
pBK-VC0998	VC0998 of WT in pBAD18-Kan, Km^R^	This study
pBK-VC1289	VC1289 of WT in pBAD18-Kan, Km^R^	This study
pBK-VCA0281	VCA0281 of WT in pBAD18-Kan, Km^R^	This study
pBK-VCA0658	VCA0658 of WT in pBAD18-Kan, Km^R^	This study
pBK-VCA0773	VCA0773 of WT in pBAD18-Kan, Km^R^	This study
pBK-VCA0988	VCA0988 of WT in pBAD18-Kan, Km^R^	This study
pΔVCA0281	pCVD442 with up- and downstream fragments of VCA0281 amplified from WT, Ap^R^	This study
pΔVCA0658	pCVD442 with up- and downstream fragments of VCA0658 amplified from WT, Ap^R^	This study
pCVDvpsA-phoA	pCVD442 with 3′ terminal and downstream fragments of *vpsA* amplified from WT, and *phoA* (amplified from *E. coli* SM10λ*pir*) inserted in between the first two fragments, Ap^R^	This study

**Table 2:** Oligonucleotides used in this study.

Primer name	Sequence (5′–3′)^a^
FP1^b^	GTAATACGACTCACTATAGGGCACGCGTGGTNTCGASTWTSGWGTT
FP2^b^	GTAATACGACTCACTATAGGGCACGCGTGGTNGTCGASWGANAWGAA
FP3^b^	GTAATACGACTCACTATAGGGCACGCGTGGTWGTGNAGWANCANAGA
FP4^b^	GTAATACGACTCACTATAGGGCACGCGTGGTAGWGNAGWANCAWAGG
FP5^b^	GTAATACGACTCACTATAGGGCACGCGTGGTNGTAWAASGTNTSCAA
FP6^b^	GTAATACGACTCACTATAGGGCACGCGTGGTNGACGASWGANAWGAC
FP7^b^	GTAATACGACTCACTATAGGGCACGCGTGGTNGACGASWGANAWGAA
FP8^b^	GTAATACGACTCACTATAGGGCACGCGTGGTGTNCGASWCANAWGTT
FP9^b^	GTAATACGACTCACTATAGGGCACGCGTGGTNCAGCTWSCTNTSCTT
FSP1^b^	GTAATACGACTCACTATAGGGC
FSP2^b^	ACTATAGGGCACGCGTGGT
SP1-tetR	GATTCCGACCTCATTAAGCAGC
SP2-tetR	GCTCTAATGCGCTGTTAATCACT
SP3-tetR	TTGACACTCTAGGATCCTAATT
VC0178-5′-SacI	AAAGAGCTCAACGGAGGTTACATGCCAAAT
VC0178-3′-SphI	AAAGCATCGTTACTTAAATTTGCGGGCAGG
VC0178-1	GATTTAACCTTCTTTCTCCTTCT
VC0178-2	CCTCTAAGATGTAACCTCCGTTTTCAATCCA
VC0178-3	CGGAGGTTACATCTTAGAGGAGAGTGAGAATG
VC0178-4	GTTCCAGGGAAGTGTATGCT
VC0512-5′-KpnI	GGCGGTACCTGATTGGGAGCGAATTATTA
VC0512-3′-XbaI	GTATCTAGAACATCTGTTTTTAATGACACCG
VC0512-1	CATTTAATGTCGGGAGAATAG
VC0512-2	CACCGAACAATTACATTTGATTGGTATAATAATT
VC0512-3	TCAAATGTAATTGTTCGGTGTCATTAAAAAC
VC0512-4	CTTTCCTTTATCTAGTCTGAAC
VC0845-5′-SacI	ATAGAGCTCATAGGCTTAAGATGAAGATTCG
VC0845-3′-XbaI	ATTTCTAGAGTACTAATGTTGTGTAACACTGT
VC0845-1	CTTTACTATCATTTTCTACGAGT
VC0845-2	AACTGTACTACATCTTAAGCCTATTAAACAAAAA
VC0845-3	GCTTAAGATGTAGTACAGTTATTCCCCCTAG
VC0845-4	TACCCAGACATCTCCCTTTTA
VC0998-5′-KpnI	AAAGGTACCGCCAGTCTATAGTTTTGCTAG
VC0998-3′-SphI	ATTGCATGCCTACACTCGCCCATTCAGC
VC0998-1	CTGCCTGCACTCGAAAAACT
VC0998-2	TCAGAGGGAGCTAGCAAAACTATAGACTGGC
VC0998-3	GTTTTGCTAGCTCCCTCTGATTCTTTCGAC
VC0998-4	GATAATGGGCTGAGTGCGG
VC1289-5′-SacI	GGGGAGCTCTTCGTTCCAACCTTTTGTTTG
VC1289-3′-SphI	GGTGCATGCTTACAGTTTAAAACGACGGATC
VC1289-1	CGGCACTCATAAAGGCTTC
VC1289-2	AGTGACGTCAAAGGTTGGAACGAAGGAGT
VC1289-3	TTCCAACCTTTGACGTCACTGACAAAGTC
VC1289-4	TTTTTTTACTGTTCATATACCCA
VCA0281-5′-SacI	AAAGAGCTCGTACCAAATTTTCTGATGCAAG
VCA0281-3′-SphI	AATGCATGCTCAGAGACTTAATCGCATCTTC
VCA0281-SacI-1	GATGAGCTCGTTTTCCCGGTGCGGAGATC
VCA0281-BamHI-2	ATCGGATCCCATACTCACCTTGCATCAGA
VCA0281-BamHI-3	ATCGGATCCTGATATGAGATCATAGCAACCA
VCA0281-XbaI-4	TGTTCTAGACTTTCAAGTATTCGATATGGATG
VCA0658-5′-EcoRI	AAAGAATTCAATTCACGTTTGTGTTGCC
VCA0658-3′-XbaI	AAATCTAGACTATTTTTGTGCAAACTGCTTC
VCA0658-XmaI-1	AATCCCGGGTGTTGTGCGTGACGT
VCA0658-2	TTCCTCGCGCACTTGTTATTAAAAACTCAAACT
VCA0658-3	AATAACAAGTGCGCGAGGAAGGGCATAG
VCA0658-XbaI-4	AAATCTAGAACGGATGTCGAATACAAAGG
VCA0773-5′-EcoRI	AATGAATTCTATGCAGGAGCCATCAT
VCA0773-3′-SphI	ATAGCATGCCTAAATTAGGTGCGGTAGCG
VCA0773-1	GATCGAGCAAACGCCCCG
VCA0773-2	ACCTAAATTACATGATGGCTCCTGCATAGC
VCA0773-3	AGCCATCATGTAATTTAGGTTTCAACTCAGCG
VCA0773-4	CAGCTTACCTAGCATGTTCCT
VCA0988-5′-EcoRI	AAAGAATTCACAGGAACTATTGAATGGAGC
VCA0988-3′-XbaI	AGGTCTAGATTATACTCGGCGGAACTGTG
VCA0988-1	CTTGCAGCAAACGGGTACG
VCA0988-2	GCTTTGTTTACATAAAAGAGCTCCATTCAATAGT
VCA0988-3	CTCTTTTATGTAAACAAAGCGCAAGAGCT
VCA0988-4	ATCCAACCCATGTCATTTTCTTCTCTC
vpsA-SacI-1	AAAGAGCTCCGTATTATCAACAAATTCCGGT
vpsA-2	ATGTACAAATCTATTTCGCTAAAATGTCCGC
phoA-3	AGCGAAATAGATTTGTACATGGAGAAAATAAAGT
phoA-4	CCTGATGAGTGGCGCGGTTTTATTTCAGCC
vpsA-5	AAACCGCGCCACTCATCAGGGGATGACAGA
vpsA-SalI-6	AAAGTCGACGATCAACCGCAATACAGTGG

a Restriction sites are underlined.

b Sequences originally designed by Wang et al. (2011).

### Construction of Deletion Mutants and Expression Plasmids

The isolation of chromosomal DNA, PCR reactions, purification of plasmids or PCR products, and construction of suicide or expression plasmids were carried out as described previously (Seper et al., 2011; Leitner et al., 2015; Pressler et al., 2016). QIAGEN plasmid kits were used for isolation of plasmid DNA; QIAquick® Gel extraction and QIAquick® PCR Purification kits (QIAGEN) were used for purifying DNA fragments. PCR reactions for fusion primer and nested integrated (FPNI)-PCR, subcloning, or generation of splicing by overlap extension (SOE)-PCR fragments were carried out using the Q5® High-Fidelity DNA Polymerase (New England Biolabs), while Taq DNA Polymerase (New England Biolabs) was used for all other PCRs.

Construction of in-frame deletion mutants in *V. cholerae* were carried out *via* two established methods. In case of ΔVCA0281 and ΔVCA0658, the suicide vector mutagenesis described by Donnenberg and Kaper using derivatives of pCVD442, i.e., pΔVCA0281 and pΔVCA0658 (Donnenberg and Kaper, 1991), was used. To generate pΔVCA0281 and pΔVCA0658, ~800 bp PCR fragments located up- and downstream of each gene were amplified using the oligonucleotide pairs VCA0281_SacI_1/VCA0281_BamHI_2 and VCA0658_XmaI_1/VCA0658_X_2, as well as VCA0281_BamHI_3/VCA0281_XbaI_4 r or VCA0658_X_3/VCA0658_XbaI_4, using chromosomal DNA from *V. cholerae* WT as template (Table 2). After digestion of the PCR fragments with the appropriate restriction enzyme (NEB) indicated by the name of the oligonucleotide, they were either directly ligated into SacI/XbaI-digested pCVD442 (for pΔVCA0281), or first connected by SOE-PCR and then ligated into XmaI/XbaI-digested pCVD442 (for pΔVCA0658). Unless noted otherwise, electrocompetent DH5αλpir were transformed with ligation products and Ap^R^ colonies were characterized for the correct constructs by PCR.

To obtain deletion strains, *E. coli* Sm10λpir were transformed with ΔVCA0281 or pΔVCA0658 and plasmids were conjugated into *V. cholerae*. Exconjugants were purified by Sm^R^/Ap^R^ selection. Sucrose selection was used to obtain Ap^S^ colonies, and chromosomal deletions were confirmed by PCR (data not shown). In all other cases, deletion mutants were obtained *via* the genome editing by chitin-induced natural co-transformation as described by Dalia et al. (2014). Briefly, 3 kb PCR fragments located up- and downstream of the respective target gene were amplified using the oligonucleotide pairs X_1/X_2 and X_3/X_4, in which X stands for the gene (Table 2). As X_2 and X_3 were designed to share an overlapping sequence at their 5′ ends, merging of the purified up- and downstream fragments into one 6 kb fragment was achieved through SOE-PCR (Horton et al., 1989). One to 3 μg of the resulting unselected PCR product were used for natural co-transformation and mixed with 40 ng of pUC19 for initial selection of overall transformants on LB-Ap agar; pUC19 was later cured from successful mutant clones through overnight growth in LB without Ap (Dalia et al., 2014). Chromosomal deletions were confirmed by PCR (data not shown), after which they were tested for Ap sensitivity by plating on LB-Ap plates and incubating overnight at 37°C.

The chromosomal transcriptional *vpsA*::*phoA* fusion strain of *V. cholerae* was generated by suicide vector mutagenesis using a derivative of pCVD442 (Donnenberg and Kaper, 1991). Briefly, 800 bp regions located at the 3′ end of the *vpsA* ORF and directly downstream of the *vpsA* locus were amplified by PCR using the primer pairs vps_SacI_1/vpsA_2 and vpsA_5/vpsA_SalI_6, respectively, and the *phoA* gene was generated by PCR from chromosomal DNA of SM10λ*pir* using the primers phoA_3 (which overlaps with vpsA_2) and phoA_4 (which overlaps with vpsA_5). All three fragments were joined by SOE-PCR into one final fragment of approximately 3.1 kb. This final fragment was digested with SacI/SalI, ligated into pCVD442 (originating pCVDvpsA-phoA), and the ligation product transformed into DH5αλ*pir*. Resulting colonies were tested by PCR (data not shown), and successful constructs were introduced into SM10λ*pir* by transformation and transferred *via* conjugation into *V. cholerae*, followed by sucrose selection of Ap^S^ colonies, according to Seper et al. (2011). Successful *vpsA*::*phoA* recombinants were verified by PCR (data not shown).

All plasmids allowing constitutive (over)-expression of the genes were constructed in a similar manner using the arabinose-inducible vector pBK (Guzman et al., 1995). PCR fragments of the genes of interest spanning from the Shine–Dalgarno sequence to the stop-codon were amplified using the oligonucleotide pairs A_5′_B and A_3′_B, in which A stands for the gene and B for the restriction site used (Table 2). PCR fragments were digested with the appropriate restriction enzyme (New England Biolabs) indicated by the name of the oligonucleotide and ligated into a similarly digested pBK. After transformation of DH5αλ*pir* with the ligation products, Km^R^ colonies were characterized by PCR (data not shown).

### Screening for In-Biofilm Repressed (*ibr*) Genes of *Vibrio cholerae*

To identify genes transcriptionally silenced during biofilm formation, we combined the library of the TetR-controlled recombination-based *in vivo* expression technology (TRIVET) and the biofilm setup of the recombination-based in biofilm expression technology (RIBET; Osorio et al., 2005; Schild et al., 2007; Seper et al., 2014; Cakar et al., 2018; Zingl et al., 2020), which was consequently renamed TetR-controlled recombination-based in-biofilm expression technology (TRIBET). An aliquot of each pool of the library (Cakar et al., 2018) was spread in triplicate on LB-Sm/Km/Ap plates. After O/N incubation ~5,000 colonies were collected from each plate, resuspended in LB-Km broth, adjusted to an OD_600_ of 2 (approximately 5 × 10^9^ CFU) and used to inoculate the reservoir in the dynamic biofilm system.

To ensure that the gene-*tnpR* fusions which we later identify as transcriptionally repressed during biofilm formation do not originate from genes induced in the planktonic phase of the reservoir, but rather in the process of biofilm formation, we used Km in the reservoir. Seven milliliter of the inoculum were added to a 50-ml conical tube, which was fixed on a metal stand with a 45° angle; we kindly refer to the original manuscript for details on the biofilm setup (Seper et al., 2014). Briefly, a cover slip (borosilicate) with a sterile P1000 pipette tip glued to one end was put in the conical tube. After a 1-h adaptation phase, LB-Sm was pumped through sterile silicon tubing (1.5 × 3 mm, VWR) with a peristaltic pump (Watson-Marlow 205S) from the supply bottle into the 1 ml tip (2 rpm, equivalent to a flow rate of approximately 10 ml/h). A hole (1 × 2 cm in dimensions) punched into the conical tube at the 5 ml mark allowed constant draining of the overflow medium and consequently kept the reservoir volume constant. Visible biofilm formed on the cover slip along the single stream of medium during a 24 h period of incubation at RT.

To remove planktonic and loosely attached cells, the flow rate was turned to maximum for 5 s before the biofilm was harvested. At 1–2 cm above the reservoir, approximately 1 cm length of biofilm was removed from the cover slip and suspended in LB. Serial dilutions were plated on LB agar lacking NaCl and supplemented with 10% Suc and Sm to select for resolved strains lacking the res or res1 cassette. After incubation for 48 h at RT, eight Suc^R^/Km^S^ colonies were picked from each biofilm output, grown in LB over night at 37°C and stored at −80°C in 96-well plates (Greiner) in LB plus 20% glycerol.

**Figure 1:**
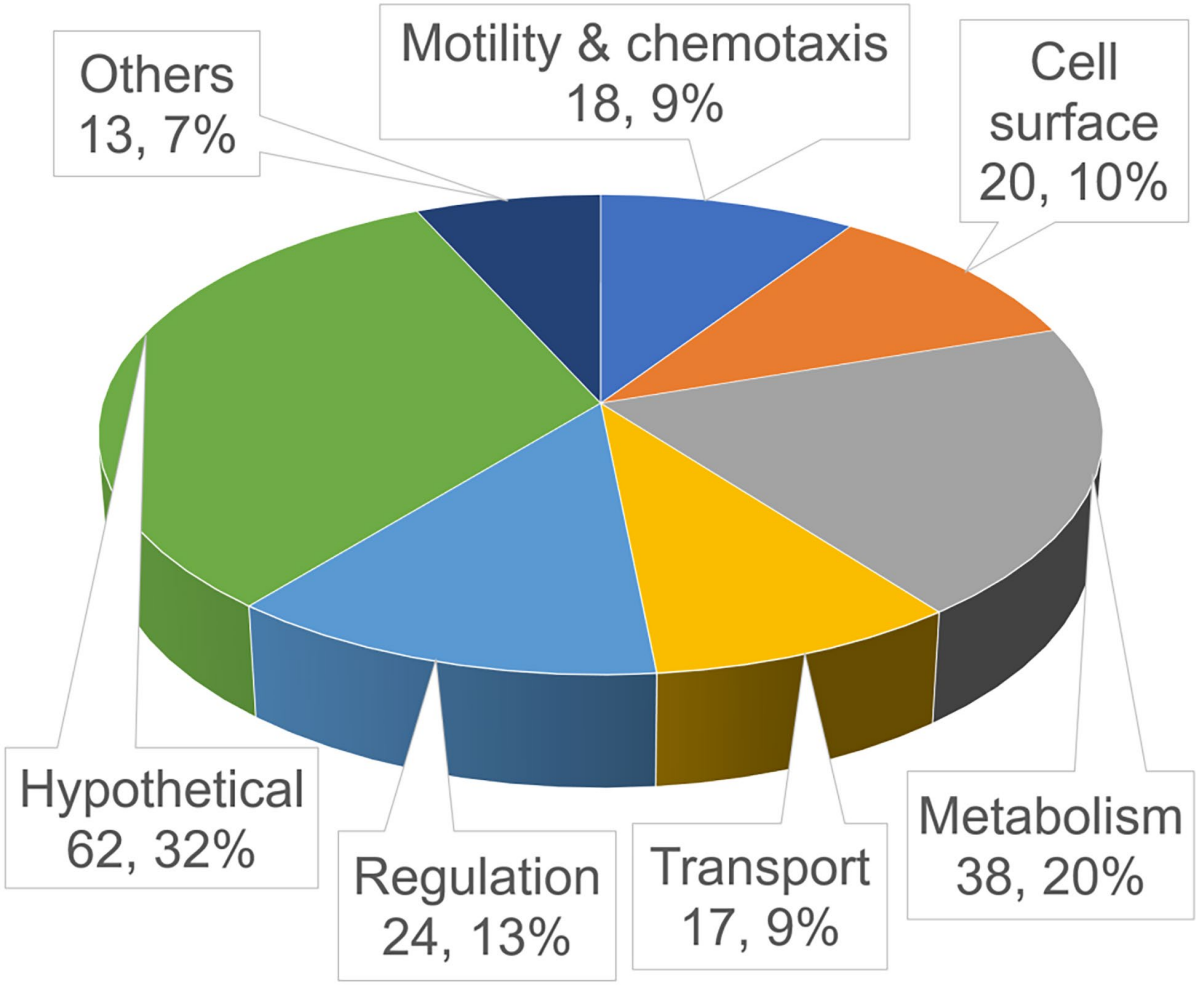
Functional distribution of the identified in-biofilm repressed (ibr) genes. Shown is a pie chart of the validated ibr genes (see also Table 3), identified with the TetR-controlled recombination-based in-biofilm expression technology (TRIBET) screening technology, allocated in functional groups by their proposed function according to Kyoto Encyclopedia of Genes and Genomes (KEGG; www.genome.jp/kegg/). The total number and percentage of ibr genes represented in the respective group are indicated by the numbers in parenthesis.

The number of false positives was reduced by quantification of the resolved TRIVET strains for PhoA activity *in vitro* as described by Cakar et al. (2018). As reported previously (Cakar et al., 2018), reanalysis of the combined data acquired by previous resolvase-based screens (Osorio et al., 2005; Schild et al., 2007; Seper et al., 2014) reveals that strains with *in vitro* resolution frequencies below 30% have only a 5% chance to be false positives. As the *tpc*-cassette remains stably integrated in the chromosome, PhoA activity of in biofilm resolved strains can be measured under *in vitro* conditions. As described previously, we used the *irgA*-fusion test strain as control to correlate the obtained PhoA activity to a resolution frequency (Cakar et al., 2018). All of the identified in biofilm resolved strains (Figure 1; Table 3) were subjected to this validation step and exhibited an *in vitro* PhoA activity higher than the *irgA*-fusion test strain as control (for details see Cakar et al., 2018).

**Table 3:** In-biofilm repressed genes of *Vibrio cholerae.*

Operon^a^	Gene locus^b^	Annotation/gene symbol^b^	No of hits^c^
VC0012-15	VC0014	DNA replication and repair protein RecF	1
VC0027-31	VC0027	Threonine dehydratase	1
	VC0031	Acetolactate synthase II catalytic subunit	1
	VC0074	Hypothetical protein	1
VC0078-79	VC0078	Ferritin	1
VC0143-144	VC0143	Hypothetical protein	1
	VC0156	TonB-dependent vitamin B12 receptor	1
	VC0175	Deoxycytidylate deaminase-like protein	4
VC0178-181	VC0178	Patatin-like protein	4
	VC0179	Hypothetical protein; cyclic AMP-GMP synthase, DncV	2
	VC0180	Hypothetical protein	1
VC0182-185	VC0184	Hypothetical protein	7
VC0193-194	VC0194	Gamma-glutamyltranspeptidase	2
VC0206-207	VC0206	N-Acetylmuramic acid-6-phosphate etherase, MurQ,	1
	VC0228	Hypothetical protein	1
VC0233-237	VC0233	3-Deoxy-D-manno-octulosonic acid transferase	1
	VC0234	Hypothetical protein	1
	VC0240	ADP-L-glycero-D-manno-heptose 6-epimerase	1
VC0241-251	VC0241	Mannose-1-phosphate guanylyltransferase	1
	VC0242	Phosphomannomutase	1
	VC0243	GDP-mannose 4,6-dehydratase	1
	VC0245	RfbG protein	2
	VC0246	Lipopolysaccharide/O-antigen transport protein	1
	VC0249	RfbL protein	6
	VC0250	Iron-containing alcohol dehydrogenase	15
	VC0251	Acyl protein synthase/acyl-CoA reductase RfbN	7
	VC0252	Acetyltransferase	1
VC0259-260	VC0259	Lipopolysaccharide biosynthesis protein RfbV, N-acetyl-alpha-D-glucosaminyl-diphospho-ditrans,octacis-undecaprenol 3-alpha-mannosyltransferase/rhamnosyltransferase	1
	VC0260	Mannosyltransferase	2
VC0262-263	VC0262	UDP-glucose 4-epimerase	1
VC0269-270	VC0270	ROK family protein	2
	VC0308	Deoxyguanosinetriphosphate triphosphohydrolase-like protein	1
	VC0353	Hypothetical protein	1
	VC0354	FKBP-type peptidyl-prolyl cis-trans isomerase FkpA	1
	VC0383	Hypothetical protein	1
	VC0390	B12-Dependent methionine synthase	1
	VC0391	Aspartate kinase	1
VC0444-445	VC0445	Survival protein SurA, peptidyl-prolyl cis-trans isomerase SurA	1
	VC0449	Methyl-accepting chemotaxis protein	1
VC0490-492	VC0490	Hypothetical protein	3
	VC0491	Hypothetical protein	1
	VC0492	Hypothetical protein	4
	VC0512	Methyl-accepting chemotaxis protein	1
VC0513-515	VC0515	Hypothetical protein	1
	VC0537	CysM; cysteine synthase B	1
VC0538-541	VC0541	Sulfate ABC transporter ATP-binding protein, sulfate/thiosulfate transport system ATP-binding protein	2
	VC0568	Cell division protein ZapE	1
VC0578-580	VC0578	Hemolysin	1
VC0591-596	VC0594	Hemolysin	2
	VC0595	Glutamyl-Q tRNA (Asp) synthetase	1
VC0632-633	VC0632	D-alanyl-D-alanine carboxypeptidase/endopeptidase	1
VC0641-646	VC0644	Ribosome-binding factor A	1
VC0671-673	VC0671	Dinucleoside polyphosphate hydrolase	1
	VC0672	Fused phosphoenolpyruvate-protein phosphotransferase PtsP/GAF domain-containing protein	1
	VC0764	Hypothetical protein	2
	VC0824	2-Cys peroxiredoxin, thiol peroxidase, atypical 2-Cys peroxiredoxin	1
	VC0845	Accessory colonization factor AcfD	2
VC0885-886	VC0886	Hypothetical protein	1
VC0900-901	VC0901	Hypothetical protein	1
VC0917-927	VC0919	Serine acetyltransferase-like protein, serine O-acetyltransferase	1
	VC0977	Putative thioredoxin	1
	VC0998	Tfp pilus assembly protein FimV	2
	VC1010	Lactoylglutathione lyase	1
	VC1033	Zinc/cadmium/mercury/lead-transporting ATPase	1
VC1092-1095	VC1093	Oligopeptide ABC transporter permease	1
VC1118-1125	VC1118	Transcriptional regulator	1
	VC1129	Inosine-guanosine kinase, inosine kinase	1
VC1155-1156	VC1155	Response regulator	1
VC1160-1162	VC1160	Hypothetical protein	1
VC1169-VC1174	VC1173	Anthranilate synthase component II (TrpG)	1
	VC1235	Sodium/dicarboxylate symporter	1
	VC1236	PilB-like protein, peptide-methionine (R)-S-oxide reductase	1
VC1237-1239	VC1237	CobT; nicotinate-nucleotide--dimethylbenzimidazole phosphoribosyltransferase	2
	VC1252	Competence damage protein CinA	1
	VC1264	Iron-regulated protein A	1
	VC1278	MarR family transcription factor	1
	VC1289	Methyl-accepting chemotaxis protein	1
	VC1309	Ribosomal-protein-alanine acetyltransferase	2
VC1312-1313	VC1313	Methyl-accepting chemotaxis protein	1
VC1314-1316	VC1316	Chemotaxis protein CheY	2
	VC1321	Hypothetical protein	2
VC1373-1375	VC1374	Molecular chaperone DnaK-related protein	1
VC1396-1403	VC1403	Methyl-accepting chemotaxis protein	1
VC1424-1428	VC1428	PotA-putrescine/spermidine ABC transporter ATPase protein	1
VC1433-1434	VC1433	Universal stress protein UspE	1
	VC1443	Hypothetical protein	1
VC1444-1445	VC1444	Hypothetical protein	1
	VC1445	Sensor histidine kinase/response regulator	1
VC1446-1448	VC1447	RTX toxin transporter, rtxD	1
	VC1492	Hypothetical protein	1
VC1549-1553	VC1552	Glycerol-3-phosphate transporter ATP-binding subunit	1
VC1602-1605	VC1602	Chemotaxis protein, CheV	2
	VC1605	Hypothetical protein	2
	VC1612	Fimbrial biogenesis and twitching motility protein, type IV pilus assembly protein, PilF	1
	VC1620	Pseudogene	1
	VC1627	NhaA-pH-dependent sodium/proton antiporter	1
VC1628-1631	VC1628	Hypothetical protein	1
VC1637-1639	VC1639	Sensor histidine kinase	1
VC1676-1678	VC1678	Phage shock protein A (PspA)	2
	VC1697	Hypothetical protein	2
	VC1707	Hypothetical protein	1
VC1714-1717	VC1714	Cell division protein MukB, chromosome partition protein	1
	VC1718	Hypothetical protein	6
VC1766-1769	VC1766	Hypothetical protein	2
	VC1768	Hypothetical protein	1
	VC1769	DNA methylase HsdM; type I restriction enzyme M protein	1
	VC1784	Neuraminidase, sialidase	1
	VC1832	Hypothetical protein	2
	VC1911	Orotidine 5′-phosphate decarboxylase	1
	VC1931	Hypothetical protein	1
VC1956-1957	VC1956	Lytic murein transglycosylase, membrane-bound lytic murein transglycosylase B	1
VC1965-1966	VC1966	Hypothetical protein	1
	VC2037	Na^+^/H^+^ antiporter, NhaC	1
VC2058-2069	VC2067	MinD-like protein; flagellar assembly protein FlhG	2
	VC2068	Flagellar biosynthesis regulator, FlhF	2
	VC2072	Peptidase/insulinase family protein	3
VC2129-2137	VC2130	FliI-flagellum-specific ATP synthase	4
	VC2143	Flagellin (FlaD)	1
	VC2187	Flagellin (FlaC)	2
	VC2318	Hypothetical protein	1
VC2324-2325	VC2324	LysR Family transcriptional regulator	1
VC2369-2370	VC2369	Aerobic respiration control sensor protein ArcB	1
	VC2370	Sensory box/GGDEF family protein, diguanylate cyclase	1
VC2376-2377	VC2377	Glutamate synthase subunit beta, glutamate synthase (NADPH) small chain, GltD	1
VC2385-2387	VC2387	Hypothetical protein	1
	VC2421	N-Acetyl-anhydromuranmyl-L-alanine amidase	1
	VC2422	Quinolinate phosphoribosyltransferase	1
VC2513-2520	VC2520	ABC transporter ATP-binding protein, MlaF, phospholipid/cholesterol/gamma-HCH transport system ATP-binding protein	1
VC2541-2542	VC2542	UDP-N-acetylmuramate:L-alanyl-gamma-D-glutamyl-meso-diaminopimelate ligase	1
	VC2545	Inorganic pyrophosphatase, inorganic pyrophosphatase	1
VC2547-2548	VC2547	Hypothetical protein	2
	VC2557	Pseudogene	1
	VC2566	Hypothetical protein	1
	VC2600	Hypothetical protein	1
	VC2619	Para-aminobenzoate synthase component II	1
	VC2651	GpsA; NAD(P)H-dependent glycerol-3-phosphate dehydrogenase, glycerol-3-phosphate dehydrogenase	1
VC2683-2684	VC2684	Bifunctional aspartate kinase II/homoserine dehydrogenase II, bifunctional aspartokinase/homoserine dehydrogenase 2	1
VC2723-2732	VC2732	General secretion pathway protein E (GspE)	1
	VC2750	GGDEF family protein	2
VC2754-2757	VC2757	Hypothetical protein	2
	VC2760	DNA-Binding transcriptional regulator	1
	VCA0011	Transcriptional regulator MalT	1
	VCA0036	Serine/threonine transporter SstT	1
VCA0105-106	VCA0106	Hypothetical protein	1
VCA0107-120	VCA0114	Hypothetical protein	1
	VCA0115	Hypothetical protein	1
VCA0127-132	VCA0128	D-Ribose transporter ATP-binding protein, ribose transport system ATP-binding protein	1
	VCA0163	Hypothetical protein	2
	VCA0168	Pseudogene	2
	VCA0186	Hypothetical protein	1
VCA0281-282	VCA0281	Integrase	2
	VCA0308	dGTPase-like protein	1
	VCA0331	Hypothetical protein	1
	VCA0334	Hypothetical protein	1
	VCA0341	Biphenyl-2,3-diol 1,2-dioxygenase	1
VCA0350-351	VCA0351	Hypothetical protein	1
	VCA0353	Hypothetical protein	1
	VCA0388	Hypothetical protein	2
VCA0391-392	VCA0392	Antidote protein, antitoxin HigA-1	1
	VCA0395	Hypothetical protein	1
VCA0396-397	VCA0396	Hypothetical protein	2
	VCA0397	Hypothetical protein	1
	VCA0399	Hypothetical protein	2
	VCA0407	Hypothetical protein	1
VCA0422-423	VCA0423	Hypothetical protein	2
	VCA0435	Hypothetical protein	1
	VCA0463	Biphenyl-2,3-diol 1,2-dioxygenase	1
	VCA0464	Hypothetical protein	3
	VCA0466	Hypothetical protein	1
	VCA0467	Hypothetical protein	1
VCA0474-475	VCA0474	Acetyltransferase	1
	VCA0480	Hypothetical protein	1
VCA0495-496	VCA0495	Hypothetical protein	1
VCA0511-512	VCA0512	Anaerobic ribonucleotide reductase-activating protein, anaerobic ribonucleoside-triphosphate reductase activating protein, nrdG	2
VCA0513-514	VCA0514	Hypothetical protein	2
	VCA0526	Chloride channel protein, Clc family	1
VCA0565-568	VCA0566	Transcriptional regulator	1
VCA0578-580	VCA0578	Hypothetical protein	2
	VCA0658	Methyl-accepting chemotaxis protein	1
VCA0692-693	VCA0693	Preprotein translocase subunit SecD	1
	VCA0702	Iron-containing alcohol dehydrogenase, NADP-dependent alcohol dehydrogenase	1
	VCA0710	TMAO reductase system periplasmic protein, periplasmic protein TorT	1
	VCA0773	Methyl-accepting chemotaxis protein	7
	VCA0802	Hypothetical protein	1
	VCA0848	Diguanylate cyclase	1
	VCA0849	Hypothetical protein	1
	VCA0895	Chemotactic transducer-like protein	1
VCA0917-918	VCA0917	TetR family transcriptional regulator	1
	VCA0988	Methyl-accepting chemotaxis protein	1
	VCA1044	Hypothetical protein	1
VCA1072-1073	VCA1073	This region contains an authentic point mutation, causing a premature stop, and is not the result of a sequencing artifact; similar to the bifunctional protein PutA and sodium/proline importer PutP (*E. coli*); identified by sequence similarity; putative	1
	VCA1084	Toxin secretion ATP-binding protein; ATP-binding cassette, subfamily C, bacterial LapB	1

a Operon predictions are according to MicrobesOnline (http://www.microbesonline.org/).

b Gene locus and annotation/gene symbols are stated according to the Kyoto Encyclopedia of Genes and Genomes (www.genome.jp/kegg/; Kanehisa and Goto, 2000).

c Number of isolates identifying the insertion site of the *tpc* cassette in the same gene locus.

To identify the exact insertion of the *tpc*-cassette, the fusion primer and nested integrated-PCR (FPNI-PCR) technique was used (Wang et al., 2011) with a few modifications. Briefly, genomic DNA from each PhoA activity-validated in biofilm resolved TRIBET strain was used as template for three primary PCRs, each using one random combination of three of the nine fusion arbitrary degenerated primers (FP1-9) listed in Table 2, together with the specific primer 1 (SP1-tetR) which is reverse-complementary to the 5′ terminal region of *tetR* in the *tpc* cassette. Primary PCRs were programmed as short reactions with a combination of annealing cycles of low, medium, and high stringency, to maximize binding of FPs to template DNA, according to the optimized strategy by Wang et al. (2011). One microliter of the products of each primary PCR was directly used as template for a secondary PCR using the fusion-specific primer 1 (FSP1), which is homologous to the fusion region of known fixed sequence at the 5′ end of each FP, and the specific primer 2 (SP2-tetR), which is nested from SP1-tetR. Secondary PCRs were standard PCRs with high stringency annealing cycles, favoring amplification of FSP1/SP2-tetR amplicons and suppressing amplification of non-specific templates. Finally, products of each secondary PCR were diluted 1:50 in ddH_2_O, and 1 μl of each dilution was used as template for a tertiary high stringency PCR using FSP2 and SP3-tetR, which are nested from FSP1 and SP2-tetR, respectively. Dilution of the products of secondary PCRs further reduces non-specific PCR products from the primary and secondary reactions to negligible amounts, while desired specific products originally generated from a FP and the SP1-tetR continue to be exponentially amplified.

Purified PCR fragments obtained at the tertiary step of FPNI-PCR were used directly as templates in sequencing reactions (LGC Genomics, Berlin, Germany) with the oligonucleotide SP3-tetR. To identify the exact position of the *tpc*-cassette, insertion sequences were subjected to the BLAST search tool^1^ of the Kyoto Encyclopedia of Genes and Genomes (KEGG) database to the *V. cholerae* N16961 genome (Kanehisa and Goto, 2000). We considered transcriptional fusions of *tetR* to any annotated ORF, if it was inserted in the same orientation lying ≤100 bp from the RBS of *tetR* as long as no factor-independent transcriptional terminators were present (Schild et al., 2007; Cakar et al., 2018).

### Static Biofilm Assays

Static biofilms in microtiter plates were assayed by crystal violet staining as previously published (Seper et al., 2011, 2014), with some modifications. Briefly, the respective strains were grown overnight on LB-Sm or LB-Km/Glc agar plates (for plasmid containing strains), suspended in LB-Sm or LB-Km/Ara (for plasmid containing strains), adjusted to an OD_600_ of 0.001 and inoculated in a 96-well microtiter plate (U bottom, Sterilin) for 24 or 48 h at RT (22°C–24°C). Wells were subsequently rinsed using a microplate washer (Anthos Mikrosysteme GmbH, Fluido2), biofilm was stained with 0.1% (w/v) crystal violet, solubilized in 96% (v/v) ethanol, and the OD_595_ was measured (microplate reader: BMG Labtech SPECTROstar^Nano^) to quantify the amount of biofilm.

### Attachment Assays and Dynamic Flow Cell Biofilm Formation

For visualization and quantification of bacterial cell adhesion and dynamically formed biofilm, the three-channel flow cell system (DTU Systems Biology, Technical University of Denmark) with 2% LB-Sm or 2% LB-Km/Ara broth was used as described previously (Seper et al., 2011). The respective overnight cultures were adjusted to OD_600_ = 0.2, and approximately, 300 μl were inoculated per channel. After static incubation for 2 h at RT (22°C–24°C), attachment was quantified by staining or flow was initiated at a constant rate of 3 ml/h with the use of a Watson Marlow 205S peristaltic pump to allow biofilm formation for a time period of 24 h at RT. After 2-h incubation for attachment assays or 24 h for dynamic biofilm assays, approximately 250 μl SYTO® 9 solution from the Live/Dead BacLight Bacterial Viability kit (Invitrogen, diluted 1:1000 in 2% LB-Sm broth) was injected per flow cell channel to stain attached cells or biofilms at RT for 20 min. Images of attached cells or biofilms were recorded with an Inverted Microscope Eclipse Ti-E (Nikon™) using 485 nm excitation and 498 nm emission. Optical sectioning was performed in 0.5 μm steps. For visualization and processing of image data, the NIS-Elements BR software (Nikon™) was used. Quantification and morphological analysis of image stacks were performed using the computer program COMSTAT2^2^ (Heydorn et al., 2000; Vorregaard, 2008).

### Microscopical Analysis of Bacterial Cell Morphology

The respective strains were grown overnight on LB-Sm or LB-Km/Glc agar plates (for plasmid containing strains), suspended and adjusted to an OD_600_ of 0.001 in LB-Sm or LB-Km/Ara (for plasmid containing strain) and cultivated at RT (22°C–24°C) for 24 and 48 h. At the given time point, an aliquot of each culture was mixed 1:1 with SYTO® 9 solution (diluted 1:500 in LB-Sm broth or in LB-Km/Ara) and incubated at RT (22°C–24°C) for 20 min. High-resolution imaging of single cells was performed with a Leica SP8 confocal microscope (Leica Microsystems Inc., Germany), with spectral detection and a HCX PL APO 63x 1.4 NA oil immersion objective. SYTO™ 9 was excited at 488 nm and fluorescence emission was detected at 500–550 nm.

### Biofilm Dispersal Assays

In order to assess the impact of ibr gene expression in mature biofilms, the three-channel flow cell system was used, akin to the procedure described for dynamic biofilm formation, but using 2% LB-Km/Glc broth instead, for repression of the P_BAD_ promoter while biofilms develop overnight. After 16 h of incubation at RT (22°C–24°C) with constant medium flow, the medium was changed to 2% LB-Km/Ara or fresh 2% LB-Km/Glc. Medium flow resumed for 8 h, and biofilms were then stained with SYTO™ 9, visualized, and quantified as defined for dynamic biofilm analysis.

### Alkaline Phosphatase Assay

Alkaline phosphatase activities (expressed in Miller Units) for TRIBET clones with ibr gene fusions or chromosomal *vpsA*::*phoA* transcriptional fusions were determined as described previously (Seper et al., 2011), using cultures with a starting OD_600_ of 0.02 grown overnight at 37°C in LB-Sm/Ap (for TRIBET clones with ibr gene fusions) or 24°C in LB-Km/Ara (for chromosomal *vpsA*::*phoA* transcriptional fusions).

### Swim Agar Assays

Swimming ability of *V. cholerae* strains was assessed by swim agar plates [1% (w/v) tryptone; 0.5% (w/v) NaCl; and 0.3% (w/v) agar] as previously described (Moisi et al., 2009). Each strain was grown on an LB-agar plate with appropriate antibiotics/supplements overnight at 37°C. Afterward, three to four single colonies were inoculated using a sterile P10 micropipette tip into swim plates. Inoculated swim plates were then incubated at 37°C for 16 h, after which the diameter of growth of the respective strain was measured.

### Statistical Analysis

Data were analyzed using the Kruskal–Wallis test, followed by *post hoc* Dunn’s multiple comparisons. GraphPad Prism version 7 was used for all statistical analyses.

## Results

### Identification of In-Biofilm Repressed (ibr) Genes in *Vibrio cholerae*

In order to identify genes which are repressed along the course of biofilm synthesis, we applied the library of the TRIVET, recently used to identify *in vivo* repressed genes in *V. cholerae* (Cakar et al., 2018; Zingl et al., 2020), to the biofilm setup established for a previous screen to identify in biofilm induced genes (Osorio et al., 2005; Schild et al., 2007; Seper et al., 2014). The methodology used herein was consequently renamed TRIBET. Briefly, the single-cell-based technology consists of three chromosomal elements, a *tetR*-*phoA*-*cat* (*tpc*) reporter cassette, a TetR-controlled *tnpR* gene, encoding a site-specific DNA recombinase (resolvase), and the resolvase target, the res cassette conferring kanamycin resistance (Kn^R^) and sucrose sensitivity (Suc^S^). The *tpc* cassette can be randomly integrated into the chromosome *via* Tn10 mutagenesis to generate transcriptional fusions of *V. cholerae* genes to the promotorless *tetR* and *phoA*. The TetR-controlled *tnpR* gene and the res cassette are inserted at a neutral site (*lacZ* locus) of the *V. cholerae* chromosome. Reporter strains with sufficient *tetR* expression *via* the *tpc* cassette will repress *tnpR* and sustain the resistance profile (Kn^R^ and Suc^S^) provided by the res cassette. In contrast, transcriptional silencing of the chromosomal promotor controlling *tetR* expression will derepress the resolvase resulting in irreversible excision of the res cassette. This allows the identification of conditionally repressed genes *via* the altered resistance profile (Km^S^ and Suc^R^).

In total, a collection of strains in 20 independent pools with approximately 500 independent *tpc* cassette insertions per pool were allowed to form biofilms on a plastic abiotic surface, after which biofilm material was collected and resuspended in LB; serial dilutions of these suspensions were plated on LB-Sm/Suc agar to select for in biofilm resolved clones. Single colonies were picked from these plates, grown overnight in LB-Sm/Ap and stored at −80°C (see Materials and Methods for more detail).

To minimize false positives, in biofilm resolved strains were further validated by assessing their PhoA activity *in vitro* as described by Cakar et al. (2018) and in materials and methods. Chromosomal DNA preparations of 288 individual biofilm-resolved and PhoA-validated colonies were amplified by FPNI-PCR and sequenced in order to identify the exact insertion site of the *tpc* cassette and upstream located genes. In total, we identified 192 unique genes that were repressed during biofilm development and can be allocated in different functional categories, while 96 sequencing approaches revealed duplicates, i.e., insertion site of the *tpc* cassette in the same gene locus (Figure 1; Table 3). Since the herein used reporter system identifies in biofilm-repressed promoters, the table additionally indicates operon predictions for each identified ibr gene.

Genes related to metabolism make up the largest group (38 genes), enforcing the notion that basal metabolism is altered and likely downregulated when cells shift from planktonic to biofilm state. Notably, operons encoding type VI secretion system (T6SS) components (VCA0105-106 and VCA0107-120) have been identified herein to be in biofilm repressed (Table 3). T6SS has been demonstrated to impact biofilm formation in several bacteria, such as *Pseudomonas aeruginosa*, *Acinetobacter baumannii*, and *Burkholderia* spp. (Aubert Daniel et al., 2008; Schwarz et al., 2010; Moscoso et al., 2011; Pan et al., 2022). In *V. cholerae*, T6SS and biofilm formation are also connected as both negatively controlled by quorum sensing (Hammer and Bassler, 2003; Zheng et al., 2010). Furthermore, genes related to motility and chemotaxis comprise a fairly large set (18 genes), validating the intuitive belief that cells need to suppress motility in order to attach to a surface and start generating a biofilm-encased sessile community. In addition to genes related to flagellar synthesis and regulation, several ibr genes could be allocated to chemotaxis including intracellular signaling (*cheV* and *cheY*) and several methyl-accepting chemotaxis proteins (MCPs). Interestingly, the later are sharply overrepresented in this screen (eight MCP genes) when compared to similar screens previously performed of *in vivo* induced (Osorio et al., 2005; Schild et al., 2007; Seper et al., 2014; Cakar et al., 2018; Zingl et al., 2020), in biofilm induced (Seper et al., 2014) and *in vivo* repressed genes (Cakar et al., 2018). *Vibrio cholerae* has a notably high number of MCPs encoded in its genome (46 MCPs) when compared to *E. coli* (four MCPs), but functional explanations for this discrepancy are currently lacking (Boin et al., 2004; Nishiyama et al., 2012). Although *V. cholerae* encodes a high number of MCPs, they represent not more than 1% of the total genes encoded by *V. cholerae*. In contrast, more than 4% of the herein identified ibr genes fall into the MCP category reinforcing their high prevalence among ibr genes.

A recent study demonstrated that constitutive expression of *in vivo* repressed genes reduces colonization fitness in the murine model (Cakar et al., 2018). In that line, constitutive expression of ibr genes may impact biofilm development indicating a pivotal role of gene silencing during this stage of *V. cholerae*’s lifecycle. For these detailed analyses, strains constitutively (over)-expressing selected ibr genes upon presence of arabinose using the pBK vector system as well as the respective in-frame deletion strains were constructed. Although the herein used arabinose-inducible pBK vector allows a tight regulation with lower expression levels than observed for IPTG-inducible systems (Guzman et al., 1995), we cannot exclude that expression levels from the pBK system are higher than in the WT. Thus, we herein refer to the pBK system as constitutive (over)-expression. Among the nine ibr genes chosen for these comprehensive analyses, we selected five MCPs based on their high abundance among ibr genes as well as randomly selected candidates of different functional categories, i.e., two surface associated factors (VC0845 and VC0998), the phospholipase CapV (VC0178) and a putative phage integrase (VCA0281). All of these randomly selected candidates were identified at least two times in the screen (Table 3). Notably, VC0998 (HubP) is also linked to chemotactic cascades, being a landmark protein in cell pole differentiation which targets several other proteins related to chemotaxis, motility and cell wall homeostasis to the cell poles *via* their cytosolic or periplasmic modules (Yamaichi et al., 2012; Altinoglu et al., 2022).

### Constitutive (Over)-Expression of ibr Genes Affects Static Biofilm Formation

**Figure 2:**
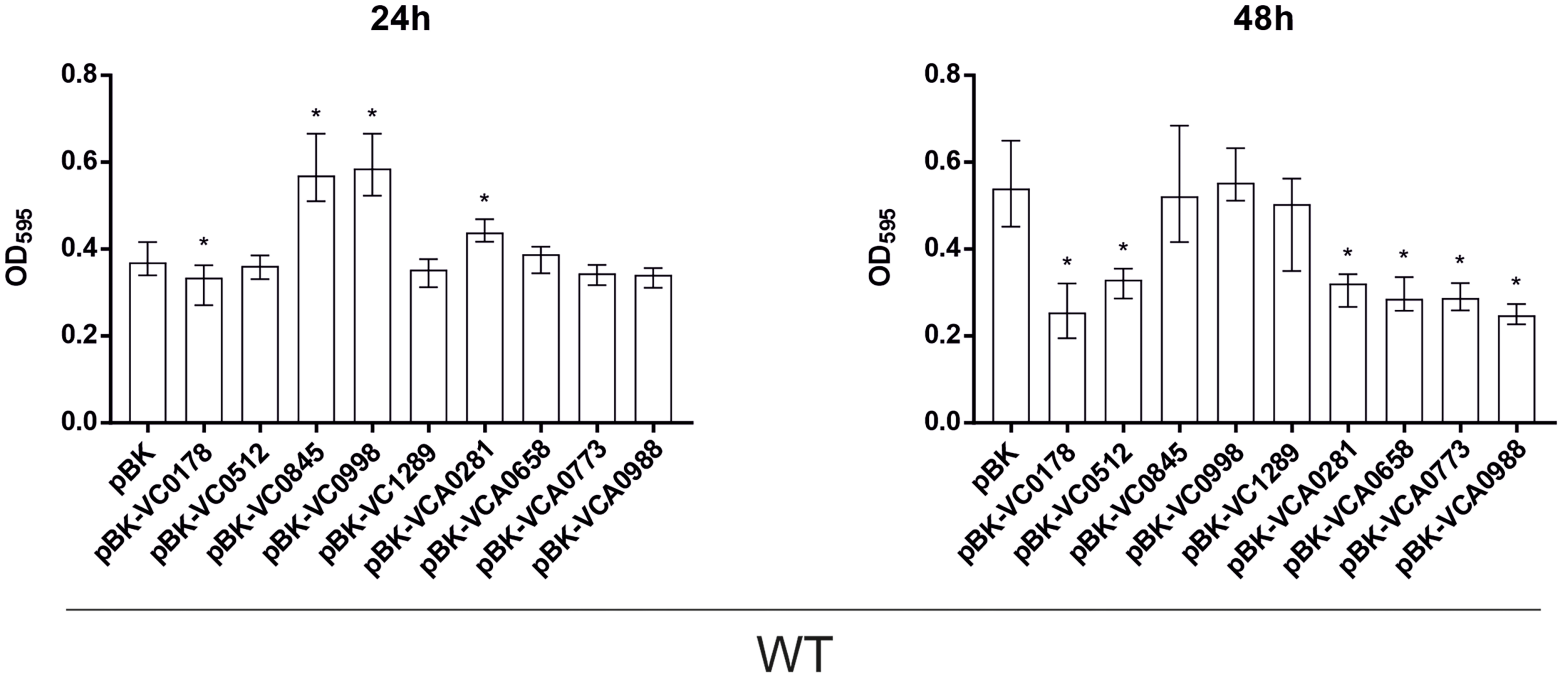
Constitutive ibr gene (over)-expression strains show an altered static biofilm formation compared to wild type (WT) with empty vector. Biofilms of WT carrying empty vector (pBK) and WT with expression plasmids of respective ibr genes, as indicated, were quantified after 24 and 48 h. The biofilm formation capacity was assayed under static conditions by crystal violet staining and subsequent determination of the OD_595_. Shown are the medians from at least 16 independent measurements. The error bars indicate the interquartile range (IQR). An asterisk indicates a significant difference to WT pBK (^*^p < 0.001 Kruskal–Wallis test followed by *post hoc* Dunn’s multiple comparisons).

**Figure 3:**
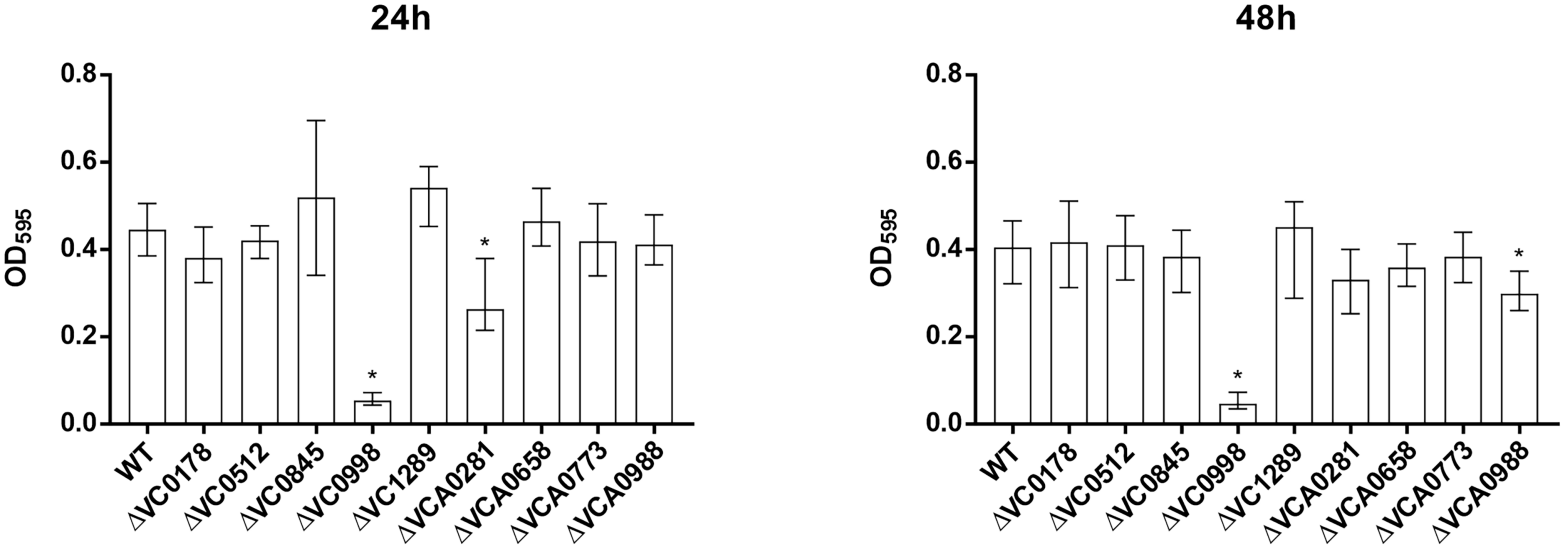
Static biofilm formation of ibr gene deletion mutants compared to the WT. Biofilms of WT and deletion mutants were quantified after 24 and 48 h. The biofilm formation capacity was assayed under static conditions by crystal violet staining and subsequent determination of the OD_595_. Shown are the medians ± IQR from at least 16 independent measurements. An asterisk indicates a significant difference to the WT (^*^p < 0.001 by Kruskal–Wallis test followed by *post hoc* Dunn’s multiple comparisons).

The aptitude for biofilm formation of each strain constitutively (over)-expressing ibr genes as well as deletion mutants was examined using static biofilm assays in microtiter plates with crystal violet staining at 24 and 48 h (Figures 2, 3). Wild type carrying the empty vector (WT pBK) served as control for the strains constitutively expressing ibr genes (Figure 2), while parental WT was used as control for the deletion mutants (Figure 3). Possible growth disparities due to constitutive (over)-expression or permanent absence of an ibr gene were monitored by OD_600_ measurements for both time points executed in parallel with the biofilm quantification (Supplementary Figures S1A, S2A). As some variations in growth dynamics were observed, the amount of biofilm detected at a given time was also normalized to the respective growth to exclude growth-dependent alterations in biofilm formation (Supplementary Figures S1A, S2A).

In general, these microtiter plate assays allow a rapid screening of diverse strains for biofilm formation. Focus herein will be given to most pronounced differences in biofilm formation capacity, although several minor, but significant alterations could be observed.

Already at 24 h, constitutive (over)-expression of VC0845 and VC0998 resulted in increased biofilm production, while constitutive (over)-expression of other ibr genes did not massively impact biofilm compared to WT pBK (Figure 2). At 48 h, WT pBK reached biofilm amounts similar to WT pBK-VC0845 and WT pBK-VC0998, while most strains constitutively (over)-expressing ibr genes (i.e., WT pBK-VC0178, WT pBK-VC0512, WT pBK-VCA0281, WT pBK-VCA0658, WT pBK-VCA0773, and WT pBK-VCA0988) showed markedly lower biofilm formation (Figure 2). Only constitutive (over)-expression of VC1289 had no significant effect on biofilm formation compared to pBK control strain (Figure 2). The majority of the deletion strains showed no or only mild alterations in biofilm formation at both timepoints compared to the parental WT (Figure 3). Notable exceptions are ΔVC0998, which shows strikingly lower biofilm levels at both 24 and 48 h, as well as VCA0281 and ΔVCA0988, which exhibit slight, but significant lower biofilm levels either at 24 or 48 h compared to the WT, respectively (Figure 3). All the observations described above are still present upon normalization for OD_600_ suggesting that growth alterations are unlikely the major cause for the observed differences in biofilm formation (Supplementary Figures S1B, S2B).

Based on the predicted function of some ibr genes in membrane composition or cell division, diverging cell morphology for ibr gene mutants or strains constitutively (over)-expressing ibr genes cannot be excluded. As such morphological alterations might affect the OD_600_, we also determined the CFU for both time points (Supplementary Figure S3). Consistent with the OD_600_ measurement only slight, but in some cases significant differences in CFU counts compared to the WT were observed. Thus, growth differences of mutants and constitutive (over)-expression strains may at best only account partially for the observed biofilm phenotypes.

Considering a predicted role of VC0998 in cell division, cell morphology of the strain constitutively (over)-expressing VC0998 and the ΔVC0998 mutant were evaluated by microscopy (Supplementary Figure S7). Comparative analyses of the mutant and WT revealed no altered cell morphology for both time points tested. Similarly, the constitutive (over)-expression strain showed no pronounced alterations in cell morphology compared to the WT carrying the empty vector at 24 h. Only at 48 h, a slight tendency toward more elongated, s-shaped cells might be observable for the strain constitutively (over)-expressing VC0998 in comparison to WT pBK (Supplementary Figure S7).

### Role of ibr Genes in Attachment, Biofilm Formation, and Detachment in a Dynamic Flow Setting

**Figure 4:**
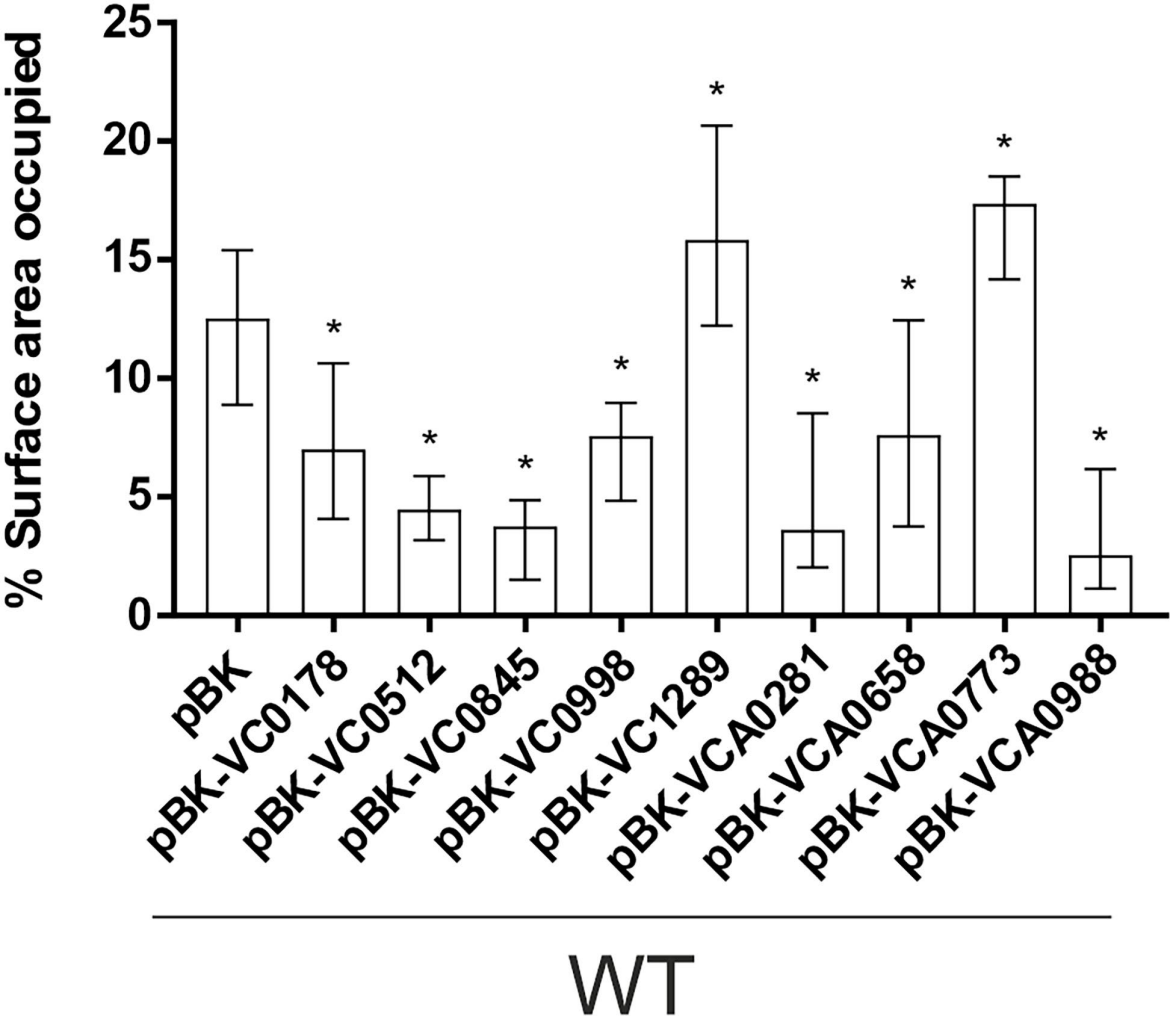
Attachment to abiotic surfaces of constitutive ibr gene (over)-expression strains is altered compared to WT with empty vector. Shown are the median percentages of surface coverage determined by the COMSTAT2 software (http://www.comstat.dk; Heydorn et al., 2000; Vorregaard, 2008) of WT with empty vector (pBK) and WT with expression plasmids of respective ibr genes, as indicated. *Vibrio cholerae* strains were allowed to attach for 2 h, before non-attached cells were removed and the attached cells were stained with SYTO™ 9. For each isolate at least eight images from four independent experiments were analyzed. Representative images are presented in Supplementary Figure S4. The error bars indicate the IQR. An asterisk indicates a significant difference to WT pBK (^*^p < 0.05 Kruskal–Wallis test followed by *post hoc* Dunn’s multiple comparisons).

Next, we investigated the efficacy of attachment and mature biofilm formation of the nine strains with constitutive (over)-expression of an ibr gene in a dynamic biofilm setup, using a three-channel flow cell system with constant medium flow (Sternberg et al., 1999). Expression of any of the nine ibr genes tested affected the ability of cells to attach to an abiotic surface compared to WT carrying the empty vector (Figure 4; Supplementary Figure S4). Constitutive (over)-expression of VC1289 and VCA0773 resulted in increased surface coverage, while all other strains constitutively (over)-expressing ibr genes showed reduced attachment.

**Figure 5:**
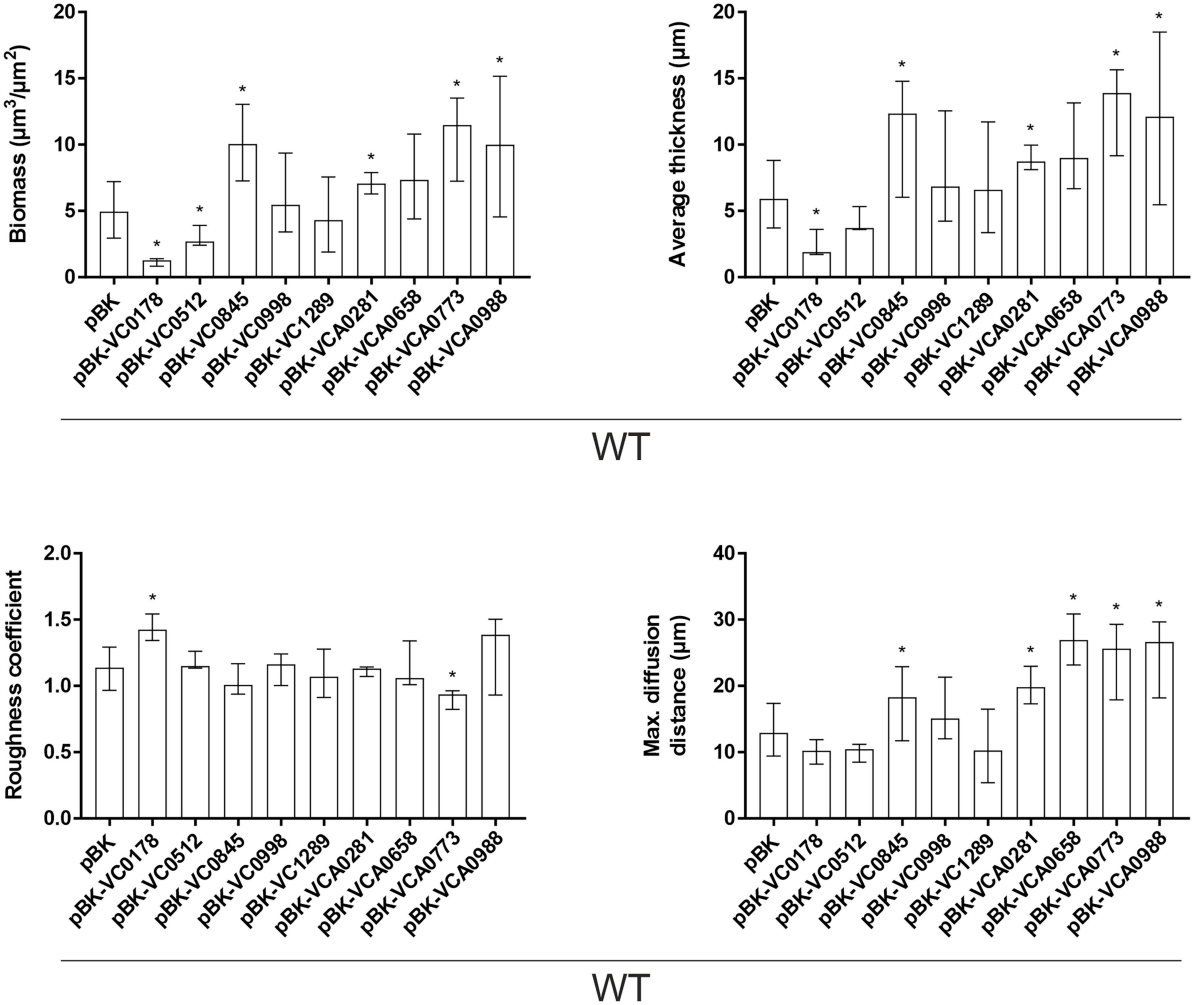
Morphological analysis of biofilms formed by constitutive ibr gene (over)-expression strains and WT with empty vector. Image stacks of WT pBK and WT with expression plasmids were analyzed for the biomass, average thickness, the roughness and maximum diffusion distance using the COMSTAT2 software (http://www.comstat.dk; Heydorn et al., 2000; Vorregaard, 2008). Biofilms were grown for 24 h in flow cell chambers with constant 2% LB medium flow, and then stained with SYTO® 9 before visualizing and processing images. Optical sectioning of the biofilms was performed in 0.5 μm steps. Representative images are presented in Supplementary Figure S5. Shown are the medians ± IQR of at least eight image stacks from two independent experiments for each strain. An asterisk indicates a significant difference to WT pBK (^*^p < 0.05 Kruskal–Wallis test followed by *post hoc* Dunn’s multiple comparisons).

These observations in attachment efficiency translated into proportional decreases or increases in biomass, average thickness, roughness and/or diffusion distances in mature biofilms where VC0178, VC0512, and VCA0773 are constitutively (over)-expressed from the start (Figure 5; Supplementary Figure S5). In contrast, decreased attachment efficiencies observed for WT pBK-VC0845 and WT pBK-VCA0281 inversely lead to increased biofilm formation under dynamic biofilm conditions indicated by elevated biomass, average thickness and diffusion distance compared to WT carrying the empty vector. Constitutive (over)-expression of VCA0658 and VCA0988 had no impact on the biomass and average thickness, but significantly increased the diffusion distance suggesting dense packaging of cell aggregates within the biofilm. Only constitutive (over)-expression of VC0998 during dynamic biofilm formation did not result in any significant change of the parameters analyzed. Notably, ΔVC0998 was the only ibr gene mutant with pronounced defect in static biofilm formation (Figure 3). Thus, we also analyzed whether the deletion mutant showed impaired surface attachment or biofilm morphology using the flow-cell setup (Supplementary Figure S6). Attachment to abiotic surfaces was not altered in ΔVC0998 compared to WT (Supplementary Figures S6A,B); however, the dynamic biofilms of the deletion mutant exhibit a different architecture, i.e., higher roughness and maximum diffusion distance, than the WT (Supplementary Figures S6C,D).

**Figure 6:**
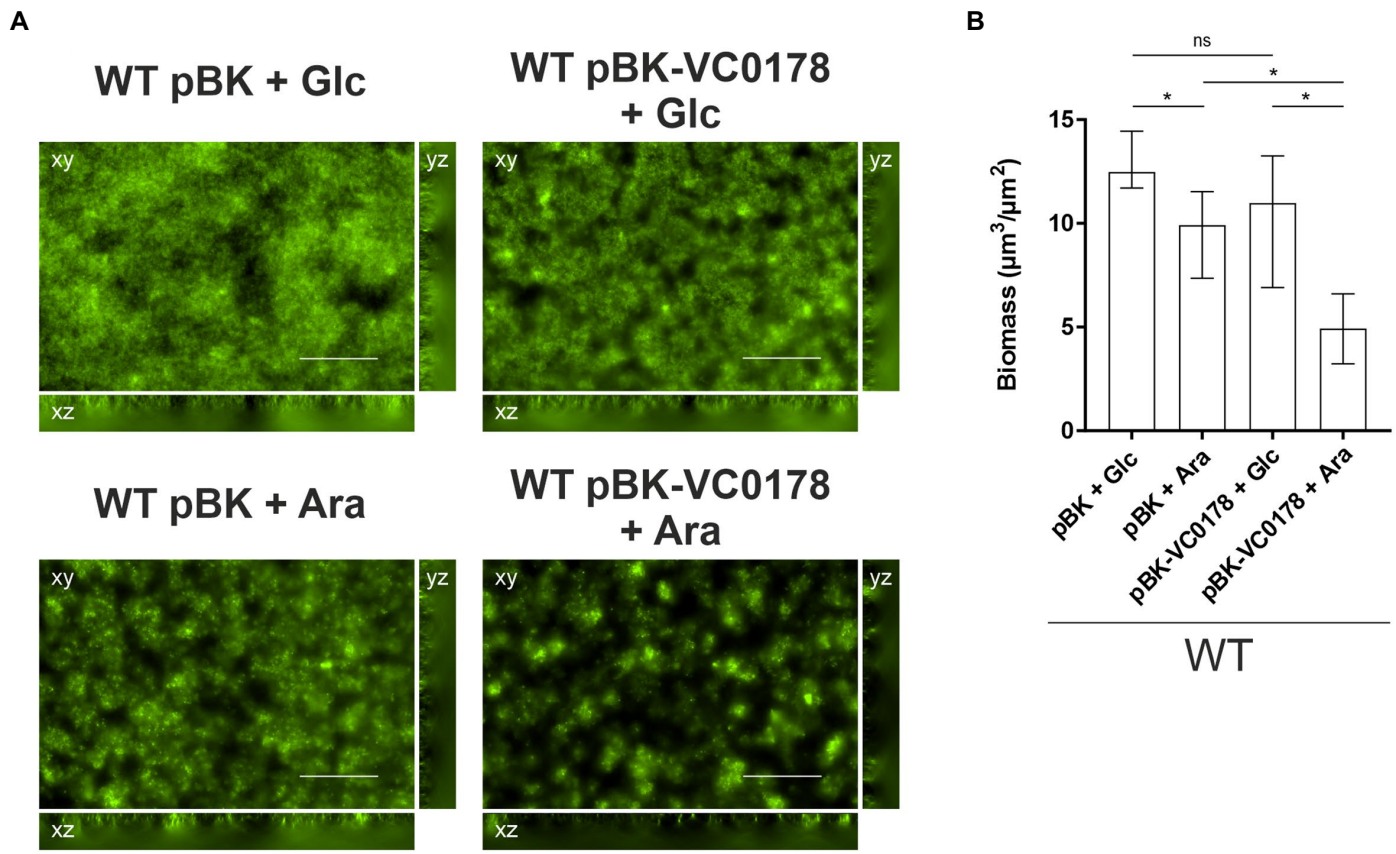
Timed induction of ibr gene VC0178 results in dispersal of mature biofilms. **(A)** Fluorescent microscopy images of SYTO™ 9 stained biofilms as horizontal (xy) and vertical (xz and yz) projections (large and side panels, respectively) of WT with empty vector (pBK) and WT with pBK-VC0178, as indicated. All biofilms were initially grown for 16 h in flow cell chambers with constant 2% LB-Km/Glc medium flow, and then for 8 more hours with constant flow of fresh 2% LB-Km/Glc medium or 2% LB-Km/Ara, as indicated above each image. Optical sectioning was performed in 0.5 μm steps. Scale bar = 50 μm. **(B)** Image stacks of WT pBK and WT pBK-VC0178 were analyzed using the COMSTAT software (http://www.comstat.dk; Heydorn et al., 2000; Vorregaard, 2008) to quantify their total biomass. Represented are the medians ± IQR of at least eight image stacks from three independent experiments for each strain. Significant differences are indicated by an asterisk (^*^p < 0.05 Kruskal–Wallis test followed by *post hoc* Dunn’s multiple comparisons).

The flow-cell setup in combination with the tight regulation of the arabinose-inducible pBAD vector system (Guzman et al., 1995) enabled us to study the impact of ibr gene induction in mature biofilms, as opposed to constitutive (over)-expression from the start of the attachment phase (see materials and methods for more detail). Briefly, biofilms were allowed to form for 16 h in presence of glucose, ensuring a tight repression of the ibr gene on the arabinose-inducible pBK vector, after which arabinose was added to trigger the expression of the ibr gene. VC0178 was selected as ibr gene candidate for this assay as: (i) throughout the study, constitutive (over)-expression of VC0178 resulted in a marked decrease of biofilm formation in static and dynamic conditions; (ii) VC0178 encodes a phospholipase, which could multiply effects upon expression due to the enzymatic activity facilitating detectability; and (iii) activity of VC0178 has been linked to alterations in the membrane composition, which could impact adhesive properties of the bacterial surface (Severin et al., 2018). No significant differences in biofilm biomass were observed between WT pBK and WT pBK-VC0178 under continuous presence of glucose (Figure 6), indicating effective glucose-mediated transcriptional silencing of VC0178. In contrast, switch from glucose to arabinose for the final 8 h resulted in significantly less biomass in biofilms of WT pBK-VC0178 compared to the WT pBK, which suggests a substantial dispersal of mature biofilms upon VC0178 expression (Figure 6). Notably, a glucose to arabinose switch for the final 8 h of biofilm formation already resulted in a slight, but significant biomass reduction and minor alterations in biofilm architecture of WT pBK. Depletion of glucose along with the arabinose supplementation likely causes metabolic and physiological changes, such as the recently reported arabinose-dependent spheroplast formation (Espinosa et al., 2020), which also might affect biofilms. Nonetheless, the glucose to arabinose switch showed much stronger effects on WT pBK-VC0178 biofilms highlighted by a biomass reduction of almost 50% and pronounced changes in morphology compared to the glucose-repressed condition (Figure 6). Thus, biofilm formation is not only inhibited by constitutive (over)-expression of VC0178 from initial stages onwards, but dispersal of mature biofilms can be also induced upon timed VC0178 expression at late biofilm stages.

### Constitutive (Over)-Expression of ibr Genes Can Interfere in Swimming Behavior

In *V. cholerae*, biofilm formation and motility are connected in several ways (Yildiz and Visick, 2009; Guttenplan and Kearns, 2013). Sensory systems, such as quorum sensing, and signaling networks revolving around the second messenger c-di-GMP, have a pivotal role in inversely regulating motility and biofilm formation. For example, higher intracellular concentrations of c-di-GMP stimulate expression of adhesion factors and extracellular matrix components while repressing motility, and vice versa (Waters et al., 2008; Conner et al., 2017; Hsieh et al., 2018). Furthermore, there is evidence that active flagellar motility is important for surface attachment (Watnick and Kolter, 1999) as well as for mature biofilm dispersal (Bridges et al., 2020).

**Figure 7:**
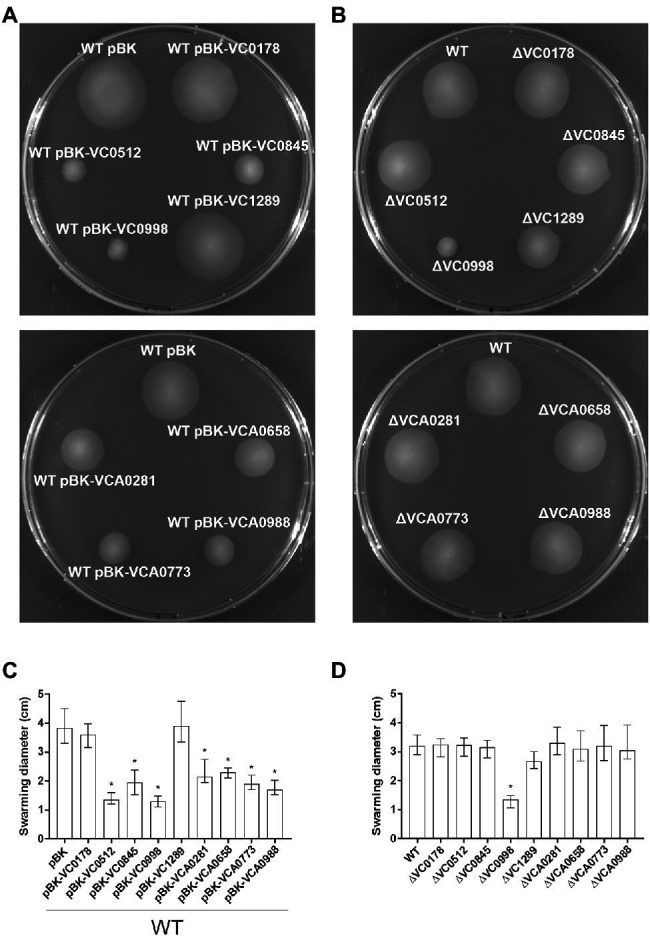
Swimming behavior of constitutive ibr gene (over)-expression strains and deletion mutants. Shown are representative swimming phenotypes of WT with empty vector (pBK) and WT with expression plasmids **(A)**, as well as of WT and deletion mutants **(B)** of respective ibr genes, as indicated, on swim agar plates. The swimming diameters of 16 independent assays for each strain are compiled in the bar chart presented as median ± IQR **(C,D)**. Significant differences between WT pBK and WT with expression plasmids, or between WT and deletion mutants, are indicated by an asterisk (^*^p < 0.001 Kruskal–Wallis test followed by *post hoc* Dunn’s multiple comparisons).

Considering the complex, yet strong, relationship between these two phenotypic aspects of *V. cholerae* as well as the overrepresentation of MCPs within the ibr genes, swimming assays in semi-solid agar were performed to reveal an impact of any of the nine ibr gene candidates (Figure 7). Constitutive (over)-expression of the MCPs VC0512, VCA0658, VCA0773, and VCA0988 showed substantially impaired swimming behavior compared to WT pBK (Figures 7A,C), while deletion of any of these genes showed no effect (Figures 7B,D). Similarly, constitutive (over)-expression of VC0845 and VCA0281 significantly decreased swimming motility, while the corresponding deletion mutant again showed no difference to the WT. Neither constitutive (over)-expression nor deletion of VC0178 and VC1289 had a significant effect on the swimming phenotype compared to WT pBK or WT, respectively. Finally, either constitutive (over)-expression or deletion of VC0998 resulted in strikingly lower swimming motility compared to their respective control strains (Figures 7A–D), suggesting that de-regulation of VC0998 in any direction impairs swimming ability.

### Impact of ibr Genes on *vpsA* Expression

**Figure 8:**
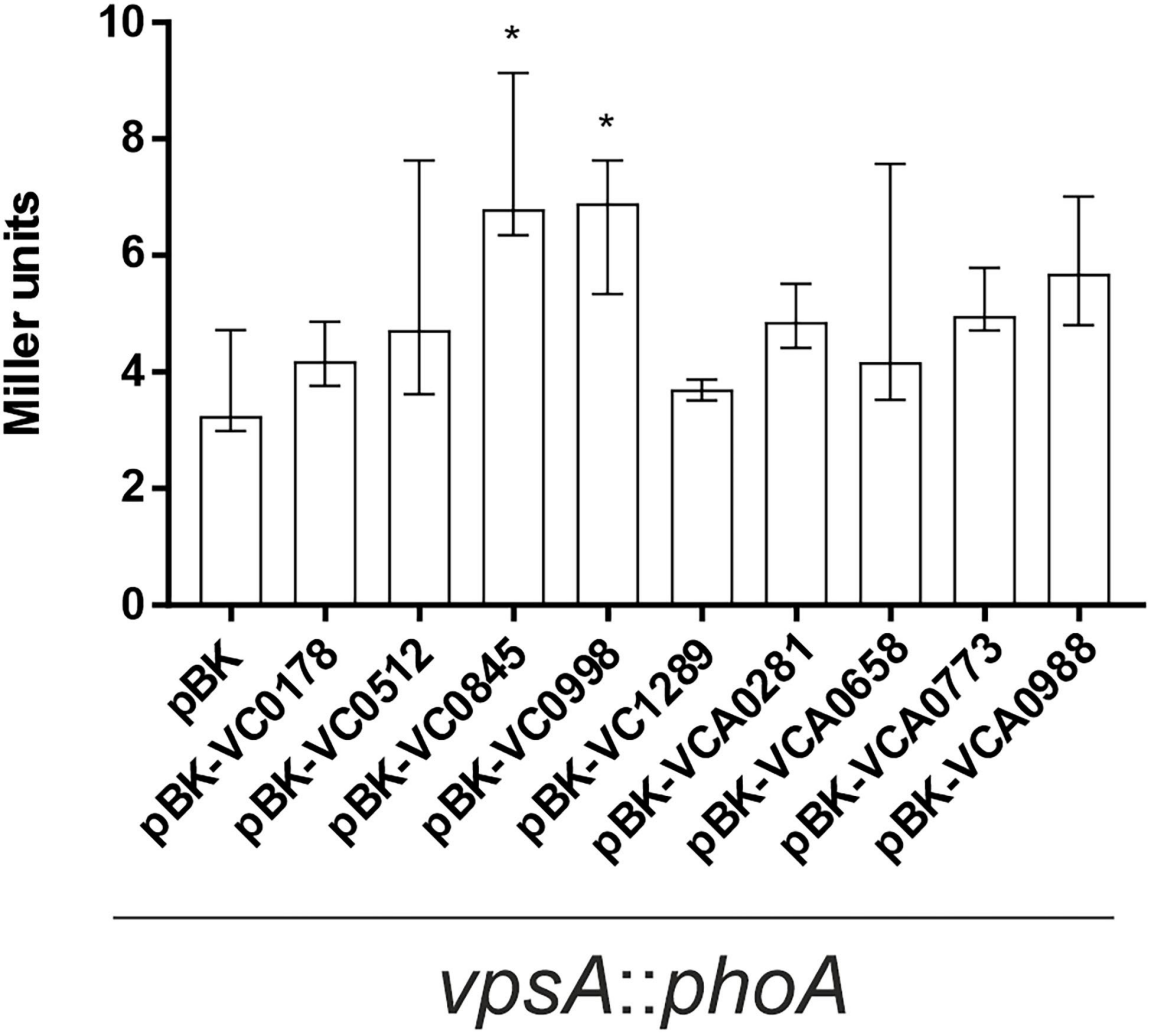
Impact of ibr gene expression on *vps* gene expression. Alkaline phosphatase activities (in Miller Units) were measured from overnight cultures of *vpsA*::*phoA* with empty vector (pBK) and *vpsA*::*phoA* with expression plasmids of respective ibr genes, at 24°C in LB-Km/Ara, as indicated. Shown are the medians ± IQR from at least eight independent measurements. An asterisk indicates a significant difference to WT pBK (^*^p < 0.001 Kruskal–Wallis test followed by *post hoc* Dunn’s multiple comparisons).

The *V. cholerae* biofilm matrix is a lattice of polysaccharides, structural proteins, extracellular DNA, and lipids (Schulze et al., 2021). The major and most prevalent component, however, is the *Vibrio* polysaccharide (VPS), a secreted substance whose synthesis and secretion are achieved by proteins encoded in 2 gene clusters, *vps*-I and *vps*-II (Fong et al., 2010). The *vps* genes are controlled by several pathways, including quorum sensing and c-di-GMP signaling, and altered *vps* gene expression directly affects biofilm formation (Yildiz and Schoolnik, 1999; Hammer and Bassler, 2003, 2009; Yildiz et al., 2004; Waters et al., 2008; Fong et al., 2010; Srivastava et al., 2011). As such, we sought to discover whether the differences in biofilm formation by strains constitutively (over)-expressing ibr genes could be linked to changes in VPS expression. To this end, a promoterless *phoA*, encoding the alkaline phosphatase (PhoA), was inserted downstream of *vpsA*, the first gene in the *vps*-I cluster (Fong et al., 2010), to generate a chromosomal transcriptional *vpsA*::*phoA* fusion. Hence, PhoA activities reflect the *vps* transcription and allowed detection of altered VPS expression levels upon ibr gene expression (Figure 8). Constitutive (over)-expression of VC0845 and VC0998 caused a significant increase in *vpsA* expression (Figure 8). This is in line with observations from static biofilm assays, where constitutive (over)-expression of VC0845 and VC0998 resulted in high static biofilm formation within 24 h (Figure 2). In addition, WT pBK-VC0845 exhibited also increased biomass compared to WT pBK in the dynamic setup (Figure 5).

## Discussion

In biofilm formation, as in any other biological process of adaptation of an individual or a group to its surroundings, a thorough modulatory program of gene expression is triggered upon recognition of specific environmental signals. In the last few decades, the genomic description of this program in *V. cholerae* has greatly expanded, mainly through generation of mutant libraries and sequencing of mutants showing aberrant biofilm behavior, in order to identify genes and genes clusters whose expression is necessary for each stage of biofilm development, spanning from initial attachment to biofilm dispersal (Yildiz and Schoolnik, 1999; Fong and Yildiz, 2007; Fong et al., 2010; Berk et al., 2012; Bridges et al., 2020). Our group has recently devised a single-cell based reporter screen for in biofilm induced genes (RIBET), which identified several new candidates relevant for biofilm formation not detected previously by conventional phenotypic screens or microarray studies (Seper et al., 2014). Still, focus has mostly been on genes necessary for biofilm formation, and which thus need to be expressed, while scarce attention has been given to genes which are unnecessary or detrimental for biofilm synthesis, but whose transcriptional silencing might be of equal importance for proper biofilm development.

With the TRIBET screen presented herein, we report a panoramic view on the biofilm repressome of *V. cholerae* including first phenotypical analyses. In total, we identified 192 genes to be in-biofilm repressed, which, together with the recently identified in biofilm induced genes (Seper et al., 2014), provides a comprehensive profile of differential gene regulation of *V. cholerae* along biofilm formation. It should be noted that these single cell-based reporter technologies rely on an irreversible excision of an antibiotic resistance cassette upon gene silencing. This allows the detection of transient gene repression and will identify spatiotemporal gene silencing of *V. cholerae* during biofilm development. It is known that several regulatory programs are temporally concerted to allow adaptation of the bacteria to the different phases of biofilm generation (Yildiz and Visick, 2009). Hence, gene expression likely varies along biofilm development, for example, genes can be repressed at one point to later be expressed, and vice versa. Indeed, four genes (VC0764, VC1612, VC2072, and VC2750) have been not only identified herein as in-biofilm repressed, but also as in biofilm induced by a previous study (Seper et al., 2014), suggesting varying induction and repression profiles along biofilm formation. Deciphering the individual spatiotemporal patterns of genes found to be differentially regulated along biofilm formation of *V. cholerae* will be a challenging task in the future.

Having such spatiotemporal patterns of gene expression in mind, strains constitutively (over)-expressing nine ibr genes as well as corresponding deletion mutants were chosen for further analyses. Constitutive (over)-expression of two ibr genes (VC0845 and VC0998) resulted in increased static biofilm formation at 24 h, while expression of five ibr genes (VC0178, VC0512, VCA0658, VCA0773, and VCA0988) reduced biofilm formation at 48 h. In comparison with the parental WT, expression of VCA0281 resulted in elevated biofilm formation at 24 h, followed by a decrease at 48 h. Thus, the majority of constitutive (over)-expression strains revealed a phenotype in static biofilm formation, while only a minority of deletion mutants exhibited significant changes, i.e., almost complete abolishment of biofilm formation for ΔVC0988 at both time points as well as slightly reduced biomass for ΔVCA0988 at 48 h. In summary, continuous expression of most ibr genes interferes in regular biofilm formation, whereas absence of ibr genes along biofilm formation can be tolerated in most cases.

Notably, constitutive (over)-expression of any of the nine ibr genes changed attachment efficacy to abiotic surfaces compared to the parental WT suggesting that presence of ibr gene products can already interfere in initial stages of biofilm development. Three strains constitutively (over)-expressing ibr genes showed the same phenotype under dynamic and static conditions (VC0178, VC0512, and VC0845). Expression of VCA0281 resulted in increased biomass in dynamic conditions, which is also consistent with the elevated static biofilm levels at 24 h. Constitutive (over)-expression of VCA0773 and VCA0988 showed inverse phenotypes in static and dynamic conditions, reinforcing the observation that requirements and molecular mechanisms of biofilm formation under these conditions can differ (Müller et al., 2007; Seper et al., 2014).

Some ibr genes and corresponding phenotypes of this work deserve further consideration. AcfD (VC0845) is one of the four accessory colonization factors of *V. cholerae*, which are transcriptionally controlled by ToxR and facilitate host colonization (Peterson and Mekalanos, 1988). The exact function of the surface associated AcfD remains to be elucidated. Repression during biofilm development is consistent with the idea that virulence and biofilm formation reflect different stages of *V. cholerae*’s life cycle and are generally inversely regulated, for example, *via* c-di-GMP (Conner et al., 2017). Constitutive (over)-expression of AcfD resulted in thick and dense biofilms with high biomass but reduced attachment to abiotic surfaces. Furthermore, constitutive (over)-expression of AcfD caused reduced motility and elevated expression of *vpsA*. Thus, AcfD might have adhesive properties facilitating inter-bacterial interactions rather than between the bacterial surface and abiotic surfaces. As such, cell–cell interaction by AcfD-decorated bacteria could interfere in swimming behavior by blocking flagellar rotation, which has been implicated as mechanosensory mechanism which triggers production and secretion of VPS upon irreversible surface attachment (Watnick et al., 2001; Lauriano et al., 2004). Alternatively, constitutive (over)-expression of AcfD could increase c-di-GMP levels, which are known to enhance VPS expression and reduction of flagellar motility (Conner et al., 2017).

Notably, ΔVC0998 was the only ibr gene mutant exhibiting a strong phenotype almost abolishing biofilm formation under static conditions. Vice versa, constitutive (over)-expression of VC0998 increased VPS expression and static biofilm formation. VC0998 (HubP) is a large transmembrane protein involved in localizing and organizing several other proteins in the cell pole, namely proteins involved in cell division, cell wall remodeling, chemotaxis and motility (Yamaichi et al., 2012; Altinoglu et al., 2022). One of the recently identified proteins requiring HubP for proper membrane localization is MotW, which has been linked to c-di-GMP degradation (Altinoglu et al., 2022). Thus, altered expression of HubP could interfere *via* MotW in c-di-GMP signaling, which in turn influences VPS expression and biofilm formation. Differential expression of HubP also impaired motility, which might also be linked to disturbances in c-di-GMP levels or other chemotaxis and motility proteins, whose polar localization is HubP-dependent (Altinoglu et al., 2022).

Constitutive (over)-expression of VC0178 consistently showed adverse effects on static and dynamic biofilm formation, while its deletion had no impact. Moreover, induction of VC0178 in mature biofilms resulted in their dispersal. To our knowledge, this is the first report of an effector, causing biofilm dissolution upon timed expression in mature biofilms. A recent study characterized VC0178 as the patatin-like phospholipase CapV, whose activity results in the release of free fatty acids from the cell membrane (Severin et al., 2018). Activity of CapV is stimulated by the second messenger 3′,3′-cyclic guanosine monophosphate-adenosine monophosphate (cGAMP), which is produced by the cGAMP synthase DncV (VC0179), also identified among the biofilm repressed genes herein (Table 3). Notably, cGAMP has been shown to downregulate biofilm formation in *E. coli* (Li et al., 2019), but so far neither cGAMP-related signaling nor CapV activity have been connected to biofilm formation in *V. cholerae*. It has been proposed that CapV remodels the cell membrane of *V. cholerae* facilitating adaptation to different environmental stressors (Severin et al., 2018).

CapV activity has not only been linked to altered membrane composition, but also to reduced membrane integrity (Severin et al., 2018). Detrimental effects on cell viability upon constitutive (over)-expression of CapV using the pBAD system are unlikely as no defect in growth or motility was observed. However, CapV activity could affect secretion of biofilm matrix components and weaken the adhesive properties of the bacterial surface, hence justifying its repression in biofilms. Interestingly, CapV belongs to the 7th pandemic island of pathogenicity 1 (VPS-1) transcriptionally controlled by the virulence regulator ToxT through the action of the small RNA TarB (Bradley et al., 2011; Davies et al., 2012), implying that CapV activity might be required during *in vivo* colonization, while being detrimental for biofilm development. Results herein show that CapV expression triggers dispersal of mature biofilms, which suggests that CapV might be induced at late biofilm stages. Indeed, a recent report highlights the induction of several virulence-related genes in biofilms, including *toxT*, facilitating hyperinfectivity of biofilm-derived *V. cholerae* (Gallego-Hernandez et al., 2020).

Finally, genes encoding for MCPs, the membrane-associated chemosensors of bacterial chemotaxis systems, are fairly overrepresented among ibr genes. No previous study using a resolvase-based single-cell reporter system to identify genes differentially regulation during intestinal colonization or genes induced during biofilm formation revealed such a high percentage of MCPs (Osorio et al., 2005; Schild et al., 2007; Seper et al., 2014; Cakar et al., 2018; Zingl et al., 2020). Strikingly, *V. cholerae* has a large number of 46 predicted MCPs when compared to closely related species such as *E. coli* (four MCPs), *Salmonella enterica* serovar Typhimurium (seven MCPs) or *Pseudomonas aeruginosa* (26 MCPs; Armitage, 1999; Boin et al., 2004; Kato et al., 2008; Hoffmann et al., 2017). The reason for this discrepancy needs yet to be elucidated, but the complex life cycle of *V. cholerae* with varying conditions might require detection of a more panoramic array of chemical signals from its environment. Such detailed sensing could facilitate acclimatization of the bacteria when transitioning between aquatic environments and the human host, between planktonic and biofilm phases, and to evolving conditions in each of these spaces and states (Nishiyama et al., 2012). Several MCPs have been identified to be induced during intestinal colonization by microarray analysis or resolvase-based screens (Osorio et al., 2005; Nielsen et al., 2006; Schild et al., 2007) and a subset has already been characterized for their roles *in vivo* (Jeffery and Koshland 1993; Everiss et al., 1994; Harkey et al., 1994; Lee et al., 2001; Nishiyama et al., 2012; Wurm et al., 2017).

The impact of MCPs on biofilm formation is less understood, most likely because they were not identified in any previous biofilm-related screen and deletion mutants, as reported herein, show no or minor effects on biofilm formation or swimming behavior. A notable exception is Mlp37, which recognizes taurine, a major constituent of bile, as an attractant (Nishiyama et al., 2016). Bile triggers various responses in *V. cholerae* including virulence induction and enhanced biofilm formation through upregulation of the *vps* genes (Hung et al., 2006). Analysis of the five ibr MCPs in this study (VC0512, VC1289, VCA0658, VCA0773, and VCA0988) provides additional insights on the impact of bacterial chemoreceptor on biofilm formation. Constitutive (over)-expression of any of the five MCPs in *V. cholerae* significantly decreased static biofilm formation and expression of four MCPs (VC0512, VCA0658, VC0773, and VCA0988) and reduced swimming ability on motility plates. The later might be explained by interference in the chemotactic response cascades downstream of the MCPs. Attachment and dynamic biofilm assays add more complexity: expression of two (VC1289 and VCA0773) increased attachment to abiotic surfaces, while expression of three (VC0512, VCA0658, and VCA0988) reduces it. Lower and higher attachment upon constitutive (over)-expression of VC0512 and VCA0773 is sustained in biomass quantification of respective dynamic biofilms. Constitutive (over)-expression of VCA0988 decreased attachment proficiency, but promoted dynamic biofilm formation.

In summary, it is evident that constitutive (over)-expression of these MCPs affects biofilm formation in different ways with diverging, detrimental effects. Thus, the identified MCPs likely exhibit individual spatiotemporal repression patterns along biofilm formation and interfere in different stages of biofilm development. In case of the last stage, a recent study implied that *V. cholerae* requires chemotaxis mediating reorientations in swimming direction to efficiently escape from mature biofilms (Bridges et al., 2020), but the required MCPs and corresponding chemotactic ligands remain to be identified. It is tempting to speculate that a complex network of differentially expressed MCPs allows transient sensing of distinct chemicals driving chemotactic responses to ensure proper biofilm development.

In conclusion, the TRIBET system successfully identified genes specifically repressed during biofilm formation, representing another successful application of reporter system besides the identification of *V. cholerae* gene repression during host passage (Cakar et al., 2018). Phenotypic analyses indicate that most ibr genes are not simply dispensable for biofilm formation, but their silencing is a necessity for ordered biofilm formation.

## Data Availability Statement

The original contributions presented in the study are included in the article/Supplementary Material; further inquiries can be directed to the corresponding author.

## Author Contributions

JPP and SS designed the study and wrote the paper. JPP, SPE, AMM, and HW performed the experiments. JPP, SPE, and SS analyzed the data. All authors contributed to the article and approved the submitted version.

## Funding

The work was supported by the Austrian Science Fund (FWF) grants: P32577 to SS, DOC50 doc.fund “Molecular Metabolism” to JPP and SS, the DocAcademy Graz to SS as well as the Land Steiermark and the City of Graz.

## Conflict of Interest

The authors declare that the research was conducted in the absence of any commercial or financial relationships that could be construed as a potential conflict of interest.

## Publisher’s Note

All claims expressed in this article are solely those of the authors and do not necessarily represent those of their affiliated organizations, or those of the publisher, the editors and the reviewers. Any product that may be evaluated in this article, or claim that may be made by its manufacturer, is not guaranteed or endorsed by the publisher.

## Footnotes

^1^ https://www.genome.jp/tools/blast/

^2^ http://www.comstat.dk
