# Supplementary material for: Impact of Gene Repression on Biofilm Formation of Vibrio cholerae
Source: Front Microbiol. 2022 Jun 2;13:912297. doi: 10.3389/fmicb.2022.912297 (PMC9201469; doi:10.3389/fmicb.2022.912297)
Supplement: Supplementary file 1 [file Data_Sheet_1.PDF]

Supplementary Material

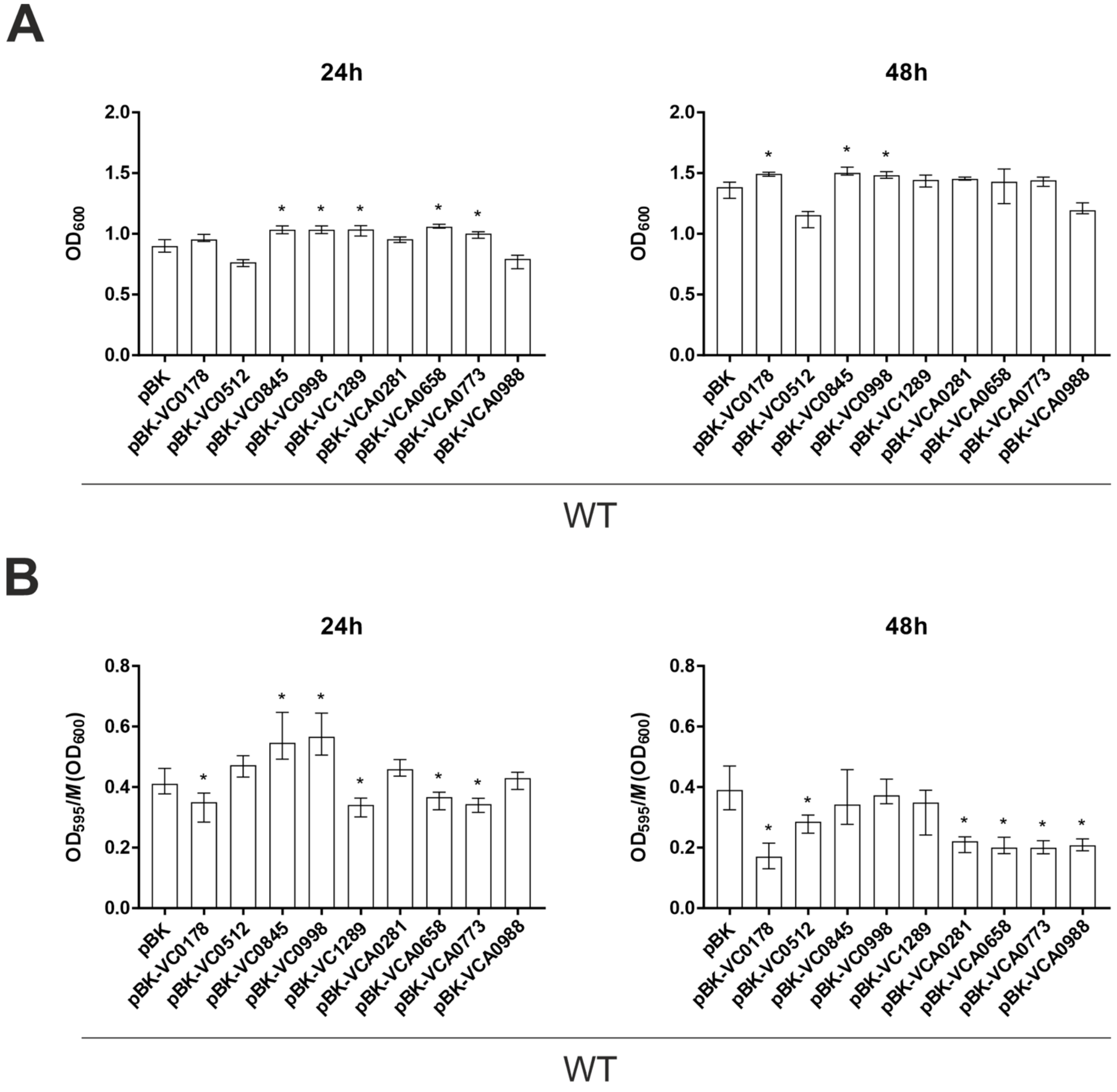

Figure S1. Growth and OD_{600}-normalized static biofilm formation of constitutive ibr gene (over)-expression strains. (A) Growth of WT carrying empty vector (pBK) and WT with expression plasmids of respective ibr genes, as indicated, were quantified after 24 h and 48 h by OD_{600} measurement. (B) OD_{595} values of static biofilm assays with WT pBK and constitutive expression strains (Figure 2) were

normalized for growth determined by OD_{600} measurement (see Material and Methods for details). (A and B). Shown are the medians ± IQR of at least 16 independent measurements. An asterisk indicates a significant difference to WT pBK (*$P < 0.001$ Kruskal–Wallis test followed by post hoc Dunn's multiple comparisons).

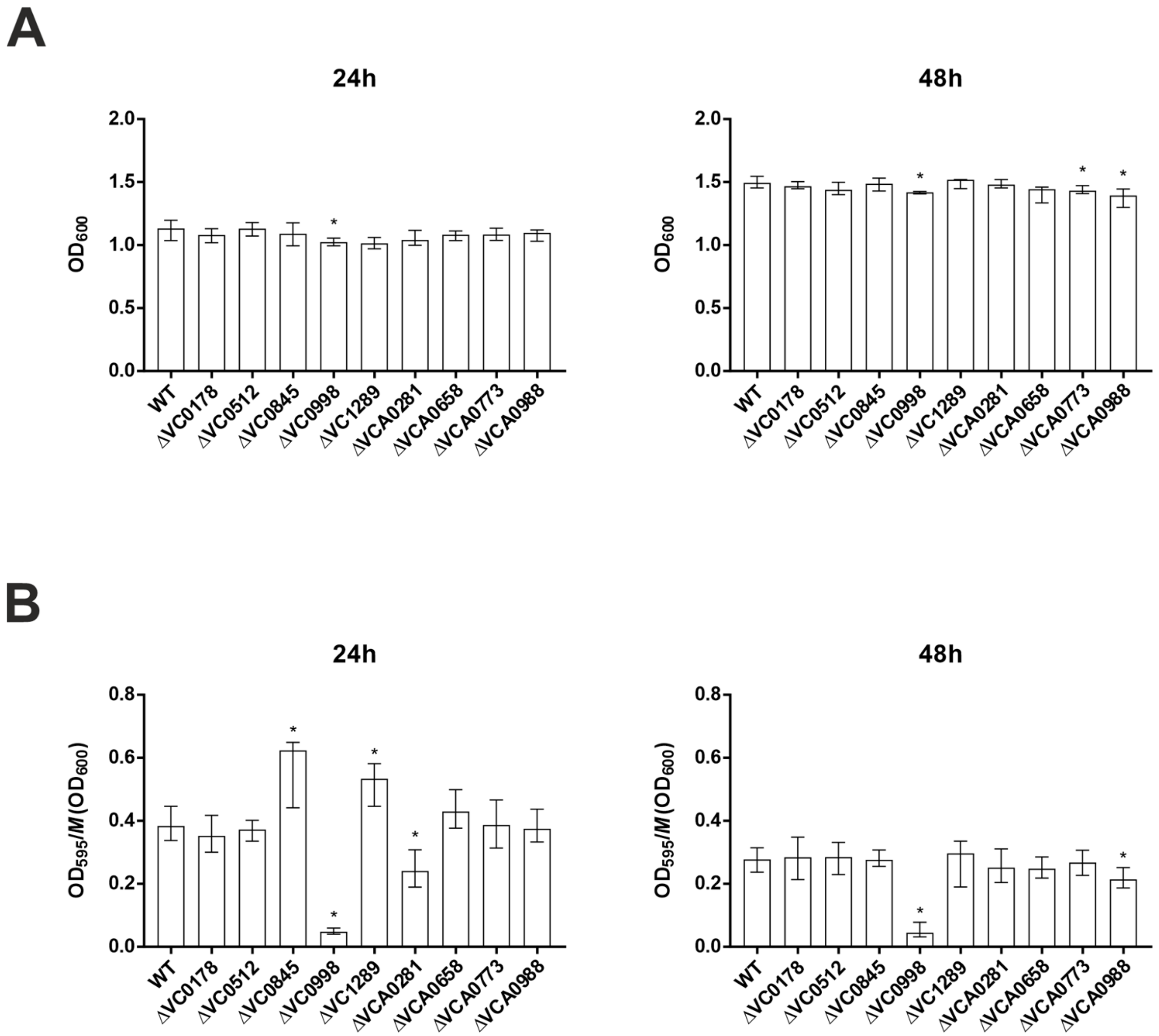

Figure S2. Growth and OD_{600}-normalized static biofilm formation biofilm formation of ibr gene deletion mutants. (A) Growth of WT and deletion mutants of respective ibr genes, as indicated, were quantified after 24 h and 48 h by OD_{600} measurement. (B) OD_{595} values of static biofilm assays with WT and deletion strains (Figure 3) were normalized for growth determined by OD_{600} measurement (see Material and Methods for details). Shown are the medians ± IQR of at least 16 independent measurements. An asterisk indicates a significant difference to the WT (*$P < 0.001$ Kruskal–Wallis test followed by post hoc Dunn's multiple comparisons).

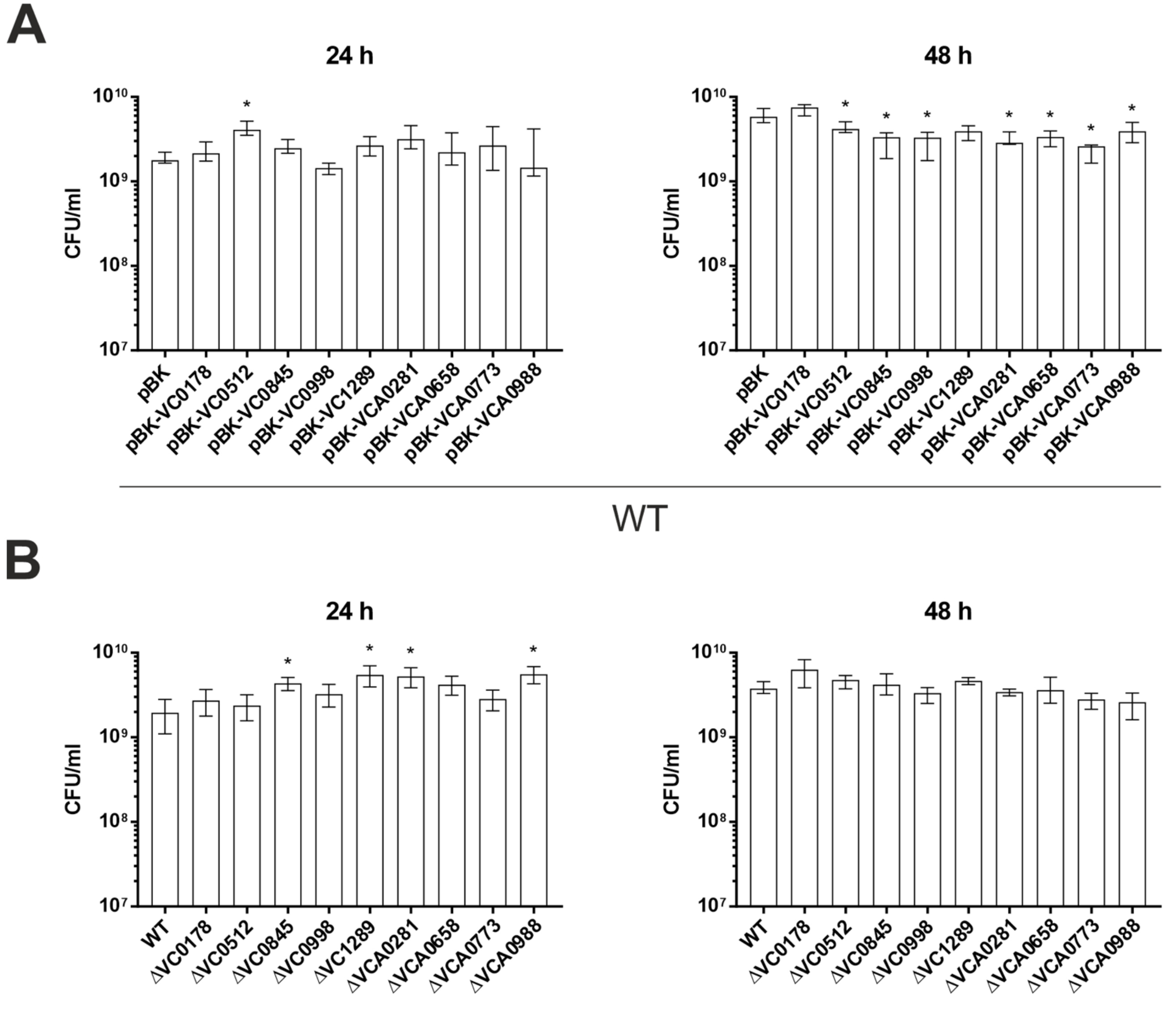

Figure S3. Comparative analyses of colony forming units (CFUs) in bacterial cultures of strains used in this study. Shown are CFU quantifications of WT with empty vector and WT with constitutive (over)-expression plasmids for respective ibr genes (A) as well as WT and ibr gene deletion strains (B). The respective strains were grown overnight on LB-Sm or LB-Km/Glc agar plates (for plasmid containing strains), suspended and adjusted to an OD_{600} of 0.001 in LB-Sm or LB-Km/Ara (for plasmid containing strain) and cultivated at RT (22 – 24°C) for 24 and 48 h. To quantify CFU appropriate dilutions of each culture were plated and grown overnight at 37°C to obtain countable colonies. Shown are the medians ± IQR of at least 7 independent measurements. An asterisk indicates a significant

difference to the WT (**P* < 0.001 Kruskal–Wallis test followed by post hoc Dunn's multiple comparisons).

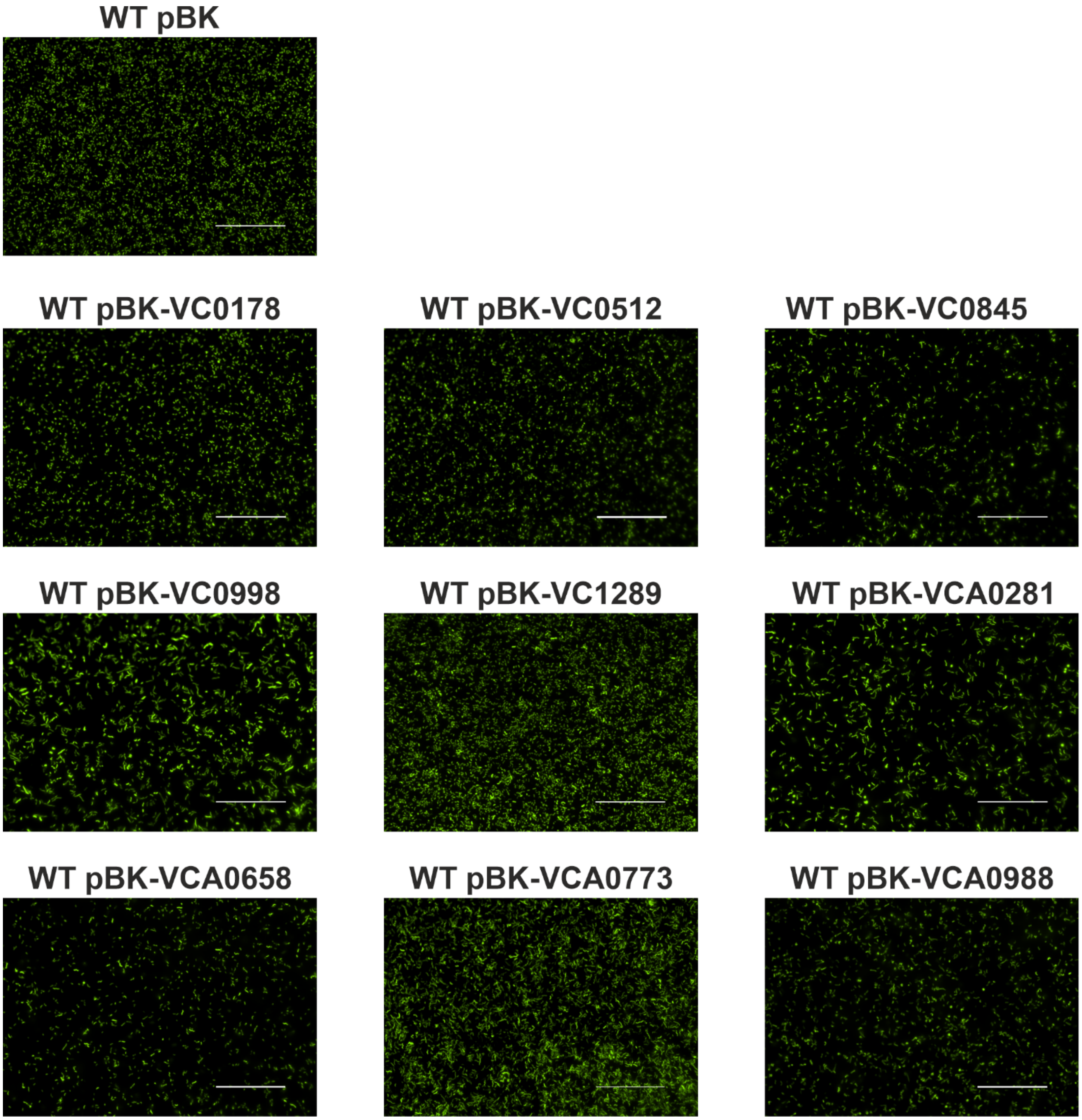

Figure S4. Visualization of surface-attached cells from constitutive ibr gene (over)-expression strains and wild type (WT) with empty vector. Shown are representative fluorescent microscopy images of SYTO^{TM} 9 stained surface-attached cells of WT with empty vector (pBK) and WT with expression plasmids of respective ibr genes, as indicated. *V. cholerae* strains were allowed to attach for 2 h, before non-attached cells were removed and the attached cells were stained with SYTO^{TM} 9.

Micrographs represent a single optical section to visualize the surface coverage on the cover slip. Scale bar = 50 μm.

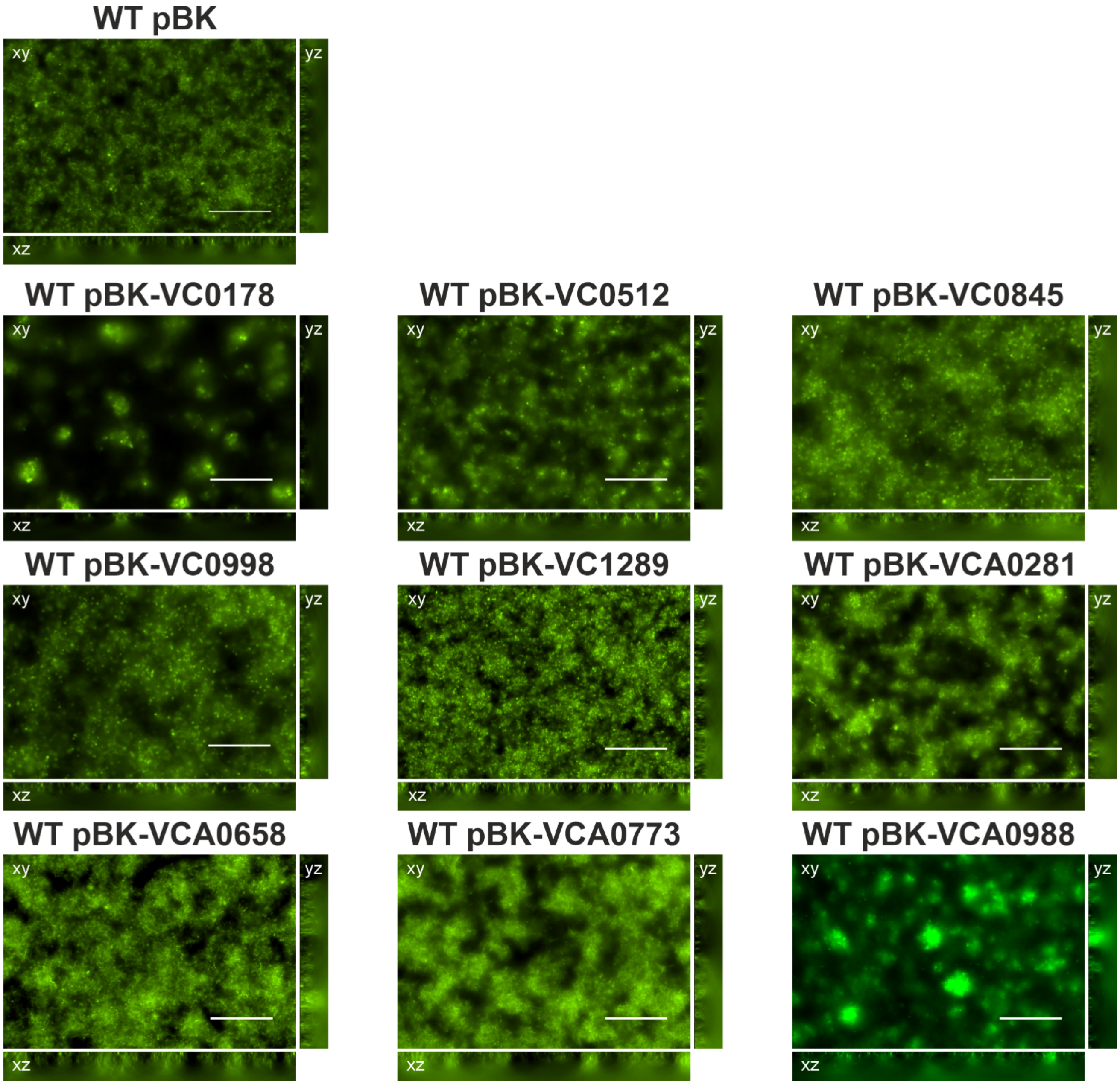

Figure S5. Visualization of three-dimensional biofilms formed by constitutive ibr gene (over)-expression strains and wild type (WT) with empty vector. Shown are representative fluorescent microscopy images of SYTO™ 9 stained biofilms as horizontal (xy) and vertical (xz and yz) projections (large and side panels, respectively) of WT with empty vector (pBK) and WT with expression plasmids of respective ibr genes, as indicated. Biofilms were grown for 24 h in flow cell

chambers with constant 2 % LB medium flow. The xy projections correspond to cross-sections at z = 15 µm. Scale bar = 50 µm.

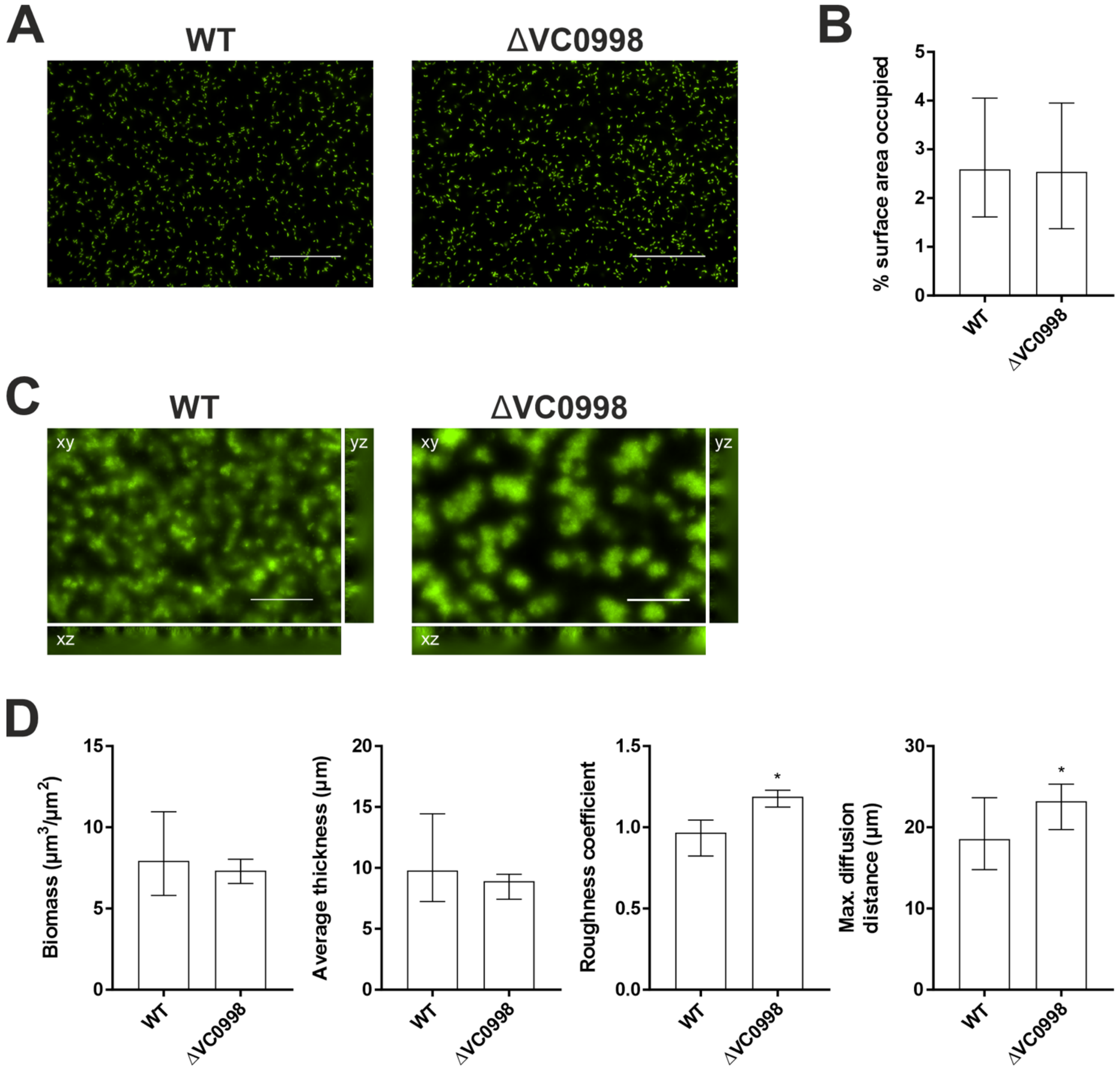

Figure S6. Microscopic analysis of surface attachment efficacy and dynamic biofilm morphology of ΔVC0998 and WT. (A, B) Shown are representative microscopic images and the median surface coverage determined by the COMSTAT2 software (http://www.comstat.dk)(Heydorn et al., 2000;Vorregaard, 2008) of WT and ΔVC0998. *V. cholerae* strains were allowed to attach for 2 h,

before non-attached cells were removed and the attached cells were stained with SYTO™ 9. Micrographs represent a single optical section to visualize the surface coverage on the cover slip. (C, D) Representative fluorescent microscopy images of SYTO™ 9-stained biofilms as horizontal (xy) and vertical (xz and yz) projections (large and side panels, respectively) of WT and ΔVC0998, and corresponding COMSTAT2 analysis of the biomass, average thickness, roughness, and maximum diffusion distance of the same strains. Biofilms were grown for 24 h in flow cell chambers with constant 2% LB medium flow. Optical sectioning was performed in 0.5 µm steps. For each isolate, at least eight images from four independent experiments were analyzed. The error bars indicate the IQR. Significant differences between WT and ΔVC0998 are indicated by an asterisk (*$P < 0.05$ Mann-Whitney test). Scale bar = 50 µm.

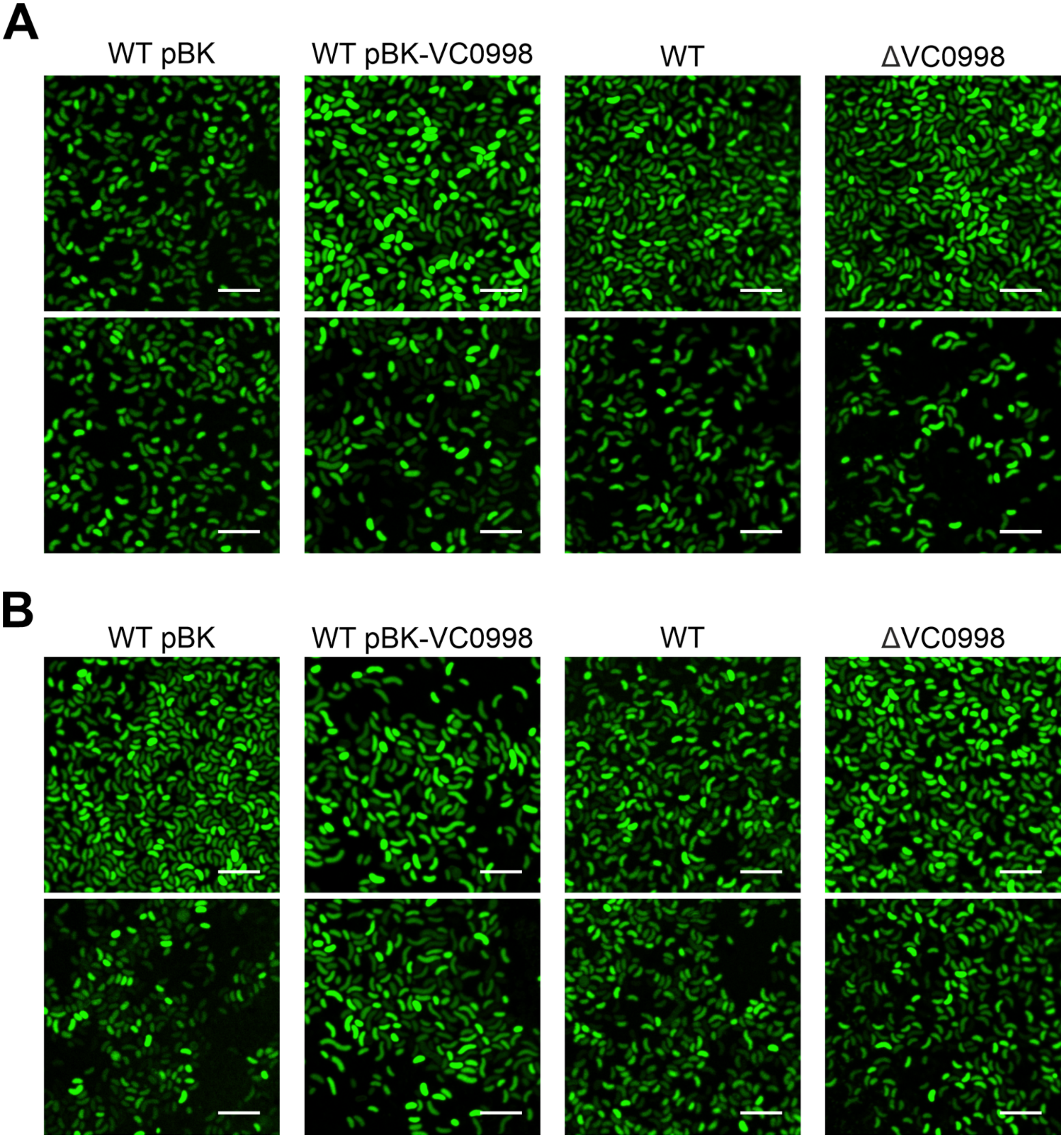

Figure S7. Analysis of cell morphology of the VC0998 (over)-expression strain, wild type (WT) with empty vector, ΔVC0998 and WT. Shown are two representative microscopic images of WT p, WT pBK-VC0998, WT and ΔVC0998. *V. cholerae* strains were grown for 24 h (A) or 48 h (B) at RT (22 – 24°C) and stained with SYTO^{TM} 9. Scale bar = 5 µm.
